# Live birth rates are unrelated to sex-steroid levels on ET day in a dydrogesterone-based ‘programmed-ovulatory FET’ protocol: a multi-centric prospective cohort study

**DOI:** 10.1093/hropen/hoaf058

**Published:** 2025-09-15

**Authors:** Tanja K Eggersmann, Noemi Hamala, Alexander R Hiller, Marion Depenbusch, Askan Schultze-Mosgau, Philippos Edimiris, Dunja Baston-Büst, Alexandra P Bielfeld, Jan-Steffen Kruessel, Sören von Otte, Wiebe Junkers, Sascha Tauchert, Reinhard Vonthein, Georg Griesinger

**Affiliations:** Department of Reproductive Medicine and Gynecological Endocrinology, University Hospital of Schleswig-Holstein, Campus Luebeck, and Universitaeres Kinderwunschzentrum Luebeck, Luebeck, Germany; Department of Reproductive Medicine and Gynecological Endocrinology, University Hospital of Schleswig-Holstein, Campus Luebeck, and Universitaeres Kinderwunschzentrum Luebeck, Luebeck, Germany; Department of Reproductive Medicine and Gynecological Endocrinology, University Hospital of Schleswig-Holstein, Campus Luebeck, and Universitaeres Kinderwunschzentrum Luebeck, Luebeck, Germany; Department of Reproductive Medicine and Gynecological Endocrinology, University Hospital of Schleswig-Holstein, Campus Luebeck, and Universitaeres Kinderwunschzentrum Luebeck, Luebeck, Germany; Department of Reproductive Medicine and Gynecological Endocrinology, University Hospital of Schleswig-Holstein, Campus Luebeck, and Universitaeres Kinderwunschzentrum Luebeck, Luebeck, Germany; Department of Obstetrics, Gynecology and REI (UniKiD), Medical Faculty and University Hospital Düsseldorf, Heinrich Heine University Düsseldorf, Düsseldorf, Germany; Department of Obstetrics, Gynecology and REI (UniKiD), Medical Faculty and University Hospital Düsseldorf, Heinrich Heine University Düsseldorf, Düsseldorf, Germany; Department of Obstetrics, Gynecology and REI (UniKiD), Medical Faculty and University Hospital Düsseldorf, Heinrich Heine University Düsseldorf, Düsseldorf, Germany; Department of Obstetrics, Gynecology and REI (UniKiD), Medical Faculty and University Hospital Düsseldorf, Heinrich Heine University Düsseldorf, Düsseldorf, Germany; Department of Reproductive Medicine and Gynecological Endocrinology, University Hospital of Schleswig-Holstein, Campus Kiel, and Universitaeres Kinderwunschzentrum Kiel, Kiel, Germany; Department of Reproductive Medicine and Gynecological Endocrinology, University Hospital of Schleswig-Holstein, Campus Kiel, and Universitaeres Kinderwunschzentrum Kiel, Kiel, Germany; Center for Reproductive Medicine, IVF-SAAR Saarbrücken-Kaiserslautern, Saarbrücken, Germany; Institute of Medical Biometry and Statistics, University of Luebeck and University Hospital of Schleswig-Holstein, Luebeck, Germany; Department of Reproductive Medicine and Gynecological Endocrinology, University Hospital of Schleswig-Holstein, Campus Luebeck, and Universitaeres Kinderwunschzentrum Luebeck, Luebeck, Germany

**Keywords:** frozen–thawed embryo transfer cycle, FET, luteal phase support, dydrogesterone, progesterone, corpus luteum, programmed-ovulatory FET, PO-FET

## Abstract

**STUDY QUESTION:**

What are the effects of three-times-a-day 10 mg oral dydrogesterone (DYD), initiated in the late follicular phase of natural menstrual cycles to induce endometrial receptivity for frozen–thawed embryo transfer (FET), on progesterone levels, indicative of ovulation on the day of FET, and how are levels of DYD, 20α-dihydrodydrogesterone (DHD), progesterone (P), and estradiol (E2) on the day of FET associated with clinical outcomes?

**SUMMARY ANSWER:**

Late follicular phase initiation of oral 30 mg DYD is compatible with progesterone levels indicative of ovulation in 98% of cases, and DYD, DHD, P, and E2 blood levels on the day of FET do not show a consistent relationship with live birth achievement, whether evaluated in isolation or interaction.

**WHAT IS KNOWN ALREADY:**

HRT regimens for FET have come under scrutiny due to: (i) the risk of insufficient progesterone exposure with conventional dosing schemes, and (ii) maternal and fetal risks associated with the iatrogenic absence of a corpus luteum. Oral DYD 10 mg three-times-a-day (tid) is considered unlikely to interfere with ovulation or corpus luteum formation and does not exhibit cross-reactivity with progesterone in ELISA. Therefore, it can be used to induce endometrial receptivity (i.e. to schedule the timing of FET in a natural cycle) and provide luteal phase support (LPS) while allowing ovulation to occur independently of the implantation window and enabling the monitoring of endogenous progesterone serum levels.

**STUDY DESIGN, SIZE, DURATION:**

Nested within a multi-centric, prospective, clinical cohort study (NCT03507673), 559 normally cycling women from the routine care population who underwent FET in a spontaneous menstrual cycle (12/2021–8/2023) had DYD, DHD, P, and E2 levels on day of FET measured by high-performance liquid chromatography/tandem mass spectroscopy (HPLC/MS/MS) or commercially available electrochemiluminescence immunoassay (ECLIA).

**PARTICIPANTS/MATERIALS, SETTING, METHODS:**

Starting from cycle day 10, women underwent endocrine (E2, LH, and P) and sonographic monitoring until the following criteria were ideally met: a leading follicle ≥16 mm, endometrial thickness ≥6 mm, and E2 ≥ 180 pg/ml. At this point, physicians could initiate oral DYD 10 mg (tid) or, if there was no LH rise in serum, postpone initiation within 2 days of monitoring to align with patient and/or centre preferences (e.g. avoiding weekends). FET for Day 2–Day 5 embryos was performed on Day 3–6 of DYD intake.

**MAIN RESULTS AND THE ROLE OF CHANCE:**

DYD and DHD plasma levels on Day 3–6 of intake replicate previous findings. In FET with Day 4 or 5 embryos, serum P levels indicated ovulation in 98.1% (>1.5 ng/ml) and 95.4% (>3.0 ng/ml) of cases and levels were higher (ratio of geometric means 2.88, 95% CI 2.48–3.35) than in FET with Day 2 or 3 embryos. Progesterone levels on day of FET were unrelated (ratio of geometric means 1.13, 95% CI 0.95–1.34) to follicular size (≤16 mm vs >16 mm) and only slightly higher (ratio of geometric means 1.36, 95% CI 1.19–1.56) in cases of serum LH level ≥12.6 IU/l versus <12.6 IU/l on day of last monitoring (i.e. before DYD initiation). When analysed stratified for FET timing (Day 2/3 vs 4/5 embryos), risk differences (RD) for live birth in subjects were only marginally and not statistically significant different in patients with low (≤25th percentile) versus normal-high (>25th percentile) hormone levels (DYD RD −5.3%, 95% CI −14.0 to 3.2, *P* = 0.227; DHD RD −4.0%, 95% CI −14.6 to 6.0, *P* = 0.428; Progesterone RD 2.9%, 95% CI −7.0 to 12.3, *P* = 0.597; E2 RD −3.3%, 95% CI −12.2 to 5.3, *P* = 0.487). These findings were confirmed across the whole range of hormone values by logistic regression, and no interaction effects of the evaluated hormones on live birth rates were found at the unadjusted significance level 0.05.

**LIMITATIONS, REASONS FOR CAUTION:**

All outcomes were assessed only in subjects undergoing FET, not on intention-to-treat. Oral DYD 10 mg (tid), similar to other progestins given to induce endometrial receptivity or for LPS, will likely still interfere with the LH surge, ovulation, and luteal phase characteristics, warranting further investigations using a true natural cycle (NC) as the reference.

**WIDER IMPLICATIONS OF THE FINDINGS:**

We propose the term ‘Programmed-Ovulatory (PO-) FET’ for this protocol. The PO-FET protocol enables scheduling the window of implantation, allows ovulation and corpus luteum formation, provides double gestagenic support, and may eliminate the need for control measurements of sex steroids on the day of FET. It allows monitoring of corpus luteum activity during the luteal phase and early pregnancy, is injection-free, and has low drug costs. This protocol needs to be tested in RCTs against other ovulatory FET protocol options, such as the ‘NC FET’ or ‘managed NC FET’.

**STUDY FUNDING/COMPETING INTEREST(S):**

The trial was funded through institutional resources of the University Hospital of Schleswig-Holstein, Campus Lübeck. Expenses related to plasma and serum sample handling, storage, shipment, and the HPLC/MS/MS and ELISA analyses of DYD, DHD, estradiol, and progesterone were financially supported by Abbott Products Operations AG (Allschwil, Switzerland). The funding was provided in the form of a research grant to the Department of Gynecological Endocrinology and Reproductive Medicine, University Hospital of Schleswig-Holstein, Campus Lübeck (no grant number applicable). Abbott Products Operations AG had no role in the study design, conduct, data collection, statistical analysis, data interpretation, manuscript preparation, or the decision to submit the article for publication. T.K.E. discloses honoraria from Ferring; travel support from Merck, Ferring, Theramex, and Gedeon-Richter; and receipt of equipment/materials (to institution) from Arthrex and Besins Healthcare, outside the submitted work. N.H. has received travel support from Gedeon-Richter, Ferring, and Merck, outside the submitted work. A.R.H. has received honoraria from Organon and travel support from Merck Serono, Gedeon Richter, and Theramex, outside the submitted work. M.D. discloses travel support from Merck, outside the submitted work. P.E. discloses honoraria from Ferring, Theramex, and Gedeon Richter; and travel support from Merck, Ferring, Theramex, Gedeon Richter, and MSD, outside the submitted work. A.P.B. discloses honoraria and travel support from Merck, Theramex, Gedeon Richter, and Ferring; and participation on a data safety monitoring board or advisory board for Ferring and Merck, outside the submitted work. A.S.-M., D.B.-B., J.-S.K., S.v.O., W.J., S.T., R.V. declare no conflict of interest. G.G. discloses consulting fees and honoraria from Merck, Organon, Ferring, Theramex, Gedeon-Richter, Abbott, Biosilu, ReprodWissen, Obseva, PregLem, Guerbet, Cooper, Igyxos, OxoLife, and ReproNovo, outside the submitted work, and travel support from Merck, Organon, Ferring, Theramex, Gedeon-Richter, and Abbott, outside the submitted work. There are no conflicts relating directly to the submitted work.

**TRIAL REGISTRATION NUMBER:**

NCT03507673.

WHAT DOES THIS MEAN FOR PATIENTS? This study explores a new method for preparing the uterus for embryo transfer in women undergoing IVF/ICSI treatments with frozen embryos. Typically, preparing the uterus (endometrium) for frozen embryo transfer (FET) involves medications to prevent ovulation (a so-called HRT protocol); however, the absence of ovulation in a conception cycle can introduce potential risks for both the mother and baby. This study evaluated an alternative to a HRT protocol by using a natural cycle FET approach, in which a low-dose oral medication called dydrogesterone (DYD) supports the uterine lining while allowing ovulation to occur naturally. For this purpose, 559 women undergoing FET in a natural menstrual cycle started DYD sometime after cycle day 10 to prepare the uterine lining. Several hormones, including progesterone and estradiol, were measured on the day of FET to determine if these levels were related to successful pregnancy outcomes. The findings showed that DYD administration most likely does not interfere with ovulation in most women, and hormone levels on the day of embryo transfer were not significantly linked to the likelihood of a successful pregnancy. This is a positive finding, as it indicates that the pregnancy is sufficiently supported by the body own hormones in combination with DYD and it also makes additional hormone monitoring unnecessary. This protocol, termed ‘Programmed-Ovulatory (PO-) FET’ may thus offer benefits by allowing a natural ovulation process and supporting the uterine lining without the need for injections and at a low financial cost. This new approach should be tested against other protocols in future studies to see if it provides better outcomes for women undergoing FET.

## Introduction

According to the latest International Federation of Fertility Societies (IFFS) report (IFFS, 2022), frozen–thawed embryo transfer (FET) cycles account for approximately 60% of all embryo transfer cycles performed globally. FET can be performed in ovulatory and anovulatory FET regimens. In anovulatory regimens (referred to as ‘HRT’ or ‘artificial’ cycles), ovulation is suppressed by estradiol administration, and the optimal time point of FET is determined by the administration of progesterone. If pregnancy ensues, early pregnancy support with exogenous sex steroids must then be extended into late first trimester (Lutjen *et al.*, 1984; Neumann *et al.*, 2020a,b). Anovulatory FET regimens have recently come under scrutiny (von Versen-Höynck and Griesinger, 2022; Magnusson *et al.*, 2024) due to: (i) the risk of suboptimal ongoing pregnancy rates caused by insufficient progesterone (P) exposure under conventional vaginal dosing schemes in a significant proportion of patients (Labarta *et al.*, 2017, 2021; Cédrin-Durnerin *et al.*, 2019; Melo *et al.*, 2021; Maignien *et al.*, 2022), and (ii) putative maternal and fetal risks resulting from the iatrogenic lack of a corpus luteum in early pregnancy (Conrad *et al.*, 2019; Ginström Ernstad *et al.*, 2019; Asserhøj *et al.*, 2021; Hu *et al.*, 2021; Moreno-Sepulveda *et al.*, 2021; Rosalik *et al.*, 2021; Busnelli *et al.*, 2022; Roelens *et al.*, 2022).

As an alternative to anovulatory FET regimens, embryo transfer can be performed during a spontaneous (i.e. natural) menstrual cycle following detection of the mid-cycle LH surge. However, relying solely on LH surge detection to time embryo transfer does not confirm ovulation or endometrial progesterone exposure, and may therefore risk asynchrony of the embryo with the window of implantation. In practice, LH monitoring requires frequent blood or urine sampling (Zaat *et al.*, 2023), increases the resource burden for both patients and clinics, and necessitates substantial organizational flexibility. Moreover, a significant proportion of cycles may still require cancellation due to unreliable detection or atypical LH profiles (Zaat *et al.*, 2023; Ho *et al.*, 2024). For this reason, a common practice is to trigger ovulation with human chorionic gonadotropin (hCG) administration when specific criteria, such as the presence of a dominant follicle on sonography, are met (the so-called managed natural cycle (NC) protocol). Although this approach can reduce the need for frequent patient monitoring to some extent, cycle cancellation remains common due to missing the LH surge or due to lack of follicular development (Groenewoud *et al.*, 2016; Ho *et al.*, 2024), Additionally, the effects of hCG administration on the luteal phase and early pregnancy endocrine characteristics and thus the time point of optimal endometrial receptivity in the context of FET have not been sufficiently studied (Tavaniotou and Devroey, 2003; Lawrenz *et al.*, 2023b). Thus, the literature remains controversial regarding whether hCG administration adversely affects pregnancy rates and live birth rates (Fatemi *et al.*, 2010; Ghobara *et al.*, 2017; Mackens *et al.*, 2020; Ho *et al.*, 2024), particularly when administered before, concomitant with, or after an endogenous LH surge (Groenewoud *et al.*, 2012; Litwicka *et al.*, 2018; Zhang *et al.*, 2023).

Recently, oral dydrogesterone (DYD) has been approved for luteal phase support (LPS) in fresh IVF cycles following extensive clinical testing in a large phase III trial program (Tournaye *et al.*, 2017; Griesinger *et al.*, 2018, 2020). An advantage of utilizing DYD is that it does not cross-react with bioidentical progesterone in commercially available, conventional ELISA, allowing for the monitoring of endogenous progesterone secretion in serum and thus ovulation without interference (Eggersmann *et al.*, 2022). Additionally, oral DYD and its primary metabolite, 20α-dihydrodydrogesterone (DHD), have been suggested to have only little interaction with the pituitary gland, thereby likely not interfering with the LH surge, spontaneous ovulation, or the activity of the corpus luteum (reviewed in Griesinger *et al.*, 2019). Thus, oral DYD could be used in normally cycling women to schedule embryo transfer without waiting for the appearance of the endogenous LH surge, while providing double gestagenic LPS (i.e. corpus luteum secretion plus administered DYD) and allowing for endocrine monitoring of ovulation and luteal phase characteristics. This study therefore investigated the use of oral DYD for FET scheduling in naturally cycling women, in what we term a ‘programmed ovulatory’ frozen–thawed embryo transfer cycle (PO-FET).

## Materials and methods

### Ethical approval

Institutional review board approval was granted (centres Luebeck & Kiel reference number 18-005; centre Duesseldorf reference number 2022-1953; centre Saarbruecken reference number 206/21), and all patients provided written informed consent.

### Design and setting

This prospective, observational, multi-centre cohort study was conducted at three university-affiliated centres and one private centre for reproductive medicine in Germany (February 2021–August 2023). It was nested within a larger platform trial (NCT03507673) investigating various aspects of endocrinology of the luteal phase and early pregnancy (Neumann *et al.*, 2020a,b, 2022) and the vaginal and endometrial microbiome (Lüth *et al.*, 2022; Depenbusch *et al.*, 2024) in women undergoing different regimens for FET.

### Study population

The present cohort consisted of 559 female patients aged 18–45 years, receiving oral DYD (10 mg tid) in an PO-FET to induce endometrial receptivity and support the luteal phase and early pregnancy. Patients, who were assumed to be ovulatory, were recruited from routine care and were undergoing FET cycles following IVF or ICSI, with no relevant uterine malformations or endometrial abnormalities (e.g. polyps), as assessed by the investigator. Patient inclusion and informed consent were obtained on Days 10–12 of a spontaneous menstrual cycle. Serum and plasma samples were collected and stored on the day of the FET (together with vaginal and endometrial swabs for separate analyses on microbial colonization). FET cycles were conducted after vitrification and warming using an open, manual system (Kitazato vitrification kit VT601, Gynemed, Lensahn, Germany; Al-Hasani *et al.*, 2007).

### PO-FET treatment regimen

**Figure 1.**
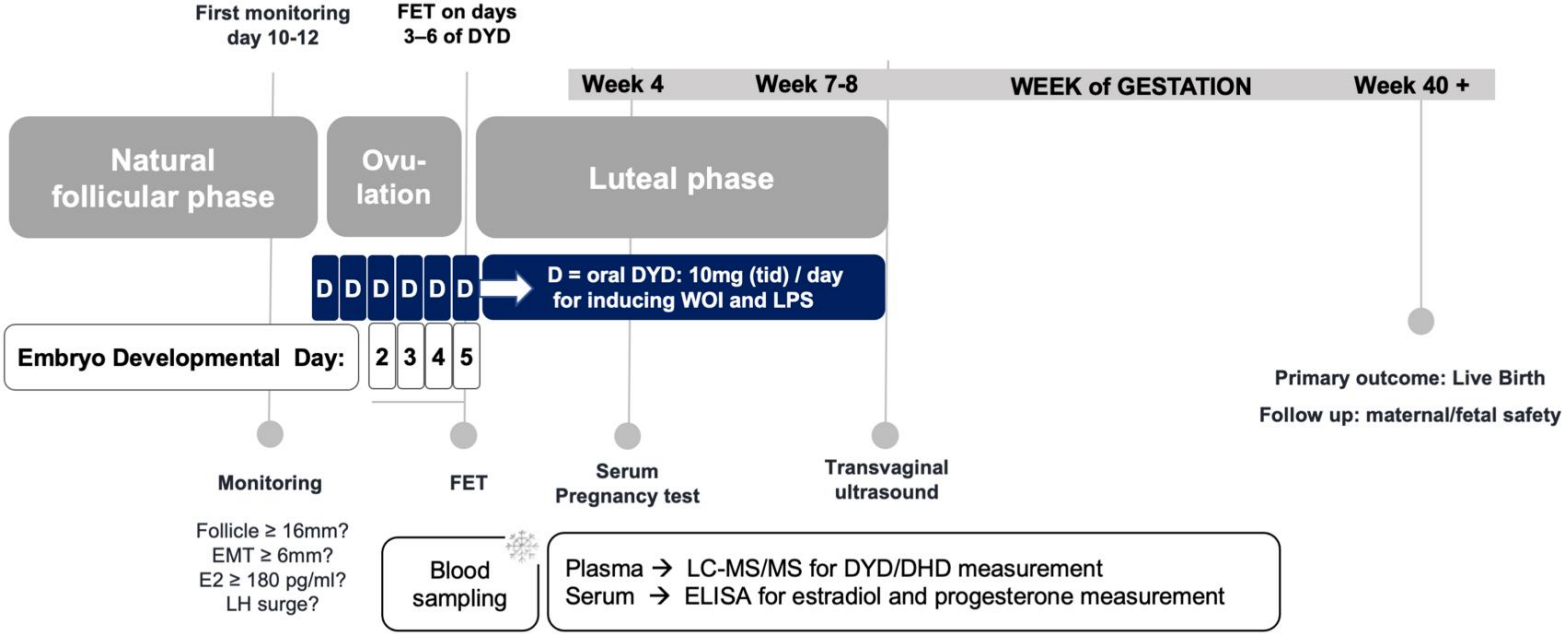
**PO-FET treatment protocol.** During the natural follicular phase (cycle days 10–12), endocrine monitoring (E2, LH, P), and transvaginal ultrasound were performed until the leading follicle reached ideally ≥16 mm, E2≥180 pg/ml, and endometrial thickness ≥6 mm. Oral dydrogesterone was initiated accordingly (DYD, 10 mg, tid). In the absence of an LH rise, DYD start could be delayed with close monitoring. Timing of frozen–thawed embryo transfer (FET) (day 3–6 of DYD) was adjusted to match the embryo developmental stage. In case of an LH surge, DYD was initiated the same or next day per physician’s judgement. Prior to FET, blood samples (serum and plasma) were collected and stored at –80°C for subsequent measurement of DYD, DHD, P, and E2. DYD was continued until clinical pregnancy confirmation (GW 7–8). The primary outcome was live birth rate. FET, frozen-thawed embryo transfer; PO-FET, progesterone-optimized frozen embryo transfer; DYD, dydrogesterone; DHD, 20α-dihydrodydrogesterone; P, progesterone; WOI, window of implantation; LPS, luteal phase support; EMT, endometrial thickness; E2, estradiol; tid, three times daily; GW, gestational week; HPLC/MS/MS, high-performance liquid chromatography/tandem mass spectrometry.

From Cycle Days 10–12, women underwent endocrine assessment of estradiol (E2), LH, and P in serum and 2D transvaginal sonographic monitoring in the morning until the leading follicle measured ≥16 mm in mean diameter, with serum E2 levels ideally reaching ≥180 pg/ml and endometrial thickness ≥6 mm in the sagittal plane. Physicians could then initiate oral DYD 10 mg (tid) on the same day. In the absence of an LH rise, DYD initiation could be delayed to the following day or postponed to a later time-point under at least 2-daily monitoring to accommodate patient and/or centre preferences (e.g. avoiding weekends). FET was performed on Days 3, 4, 5, or 6 of DYD intake, timed to match the embryo’s developmental stage: Day 3 of DYD intake for a Day 2 embryo, Day 4 of DYD intake for a Day 3 embryo, Day 5 of DYD intake for a Day 4 embryo, and Day 6 of DYD intake for a Day 5 embryo (Fig. 1). If an LH surge, as defined by the local investigator, was detected during monitoring, oral DYD administration had to be initiated the same day, or the following day if no progesterone elevation was observed, even if the above criteria were not met in combination. Of note, neither LH surges nor progesterone elevation were defined by the study protocol but were left to the judgement of the treating physician. Women who achieved pregnancy, continued oral DYD 10 mg (tid) intake until confirmation of clinical pregnancy (i.e. the 7–8th gestational week [GW]).

### Sampling and outcomes

FET was typically performed in the late morning. Before FET, blood samples (serum and plasma) were collected and stored at -80°C for later measurement of DYD, DHD, P, and E2. A vaginal swab (Copan UTM^®^: Universal Transport Medium for Viruses, Chlamydia, Mycoplasma, and Ureaplasma. Copan Diagnostics Inc., Murrieta, CA) and an aspiration of endometrial fluid (Gynectics Tulip Memo Transfer Catheter, Gynétics Medical Products N.V., Lommel, Belgium) were performed immediately before FET for microbiome analysis (separate analysis). A positive pregnancy test (i.e. serum hCG levels above the local centre’s reference range) 10–14 days after FET indicated implantation. Clinical pregnancy was defined by the presence of a fetal sac with a heartbeat on transvaginal sonography at GW 7 or later. Prospective follow-up monitoring after GW 7 consisted of tracking pregnancy progression, adverse events, live birth, and the health of the child through telephone communication by a study nurse or doctoral student. Pregnancy outcomes included maternal and fetal/neonate morbidity, termination of pregnancy, and fetal malformations assessed by prenatal diagnosis or at birth (separate analyses). Live birth, chosen as the primary outcome of interest for the present analysis, was defined as the delivery of a living infant at or beyond the point of viability. Ongoing or past ovulation was assessed by measuring serum progesterone levels on day of FET. Ovulation was assumed if progesterone exceeded 1.5 ng/ml on that day. In cases where this threshold was not reached on the day of FET, but was exceeded on the day of the pregnancy test, ovulation was also assumed. This assessment was conducted in the subgroup of patients undergoing transfer on Day 5 or Day 6 of DYD intake (i.e. morula stage and blastocyst stage), and also in the total population. Sensitivity analyses used a more conservative threshold of ≥3.0 ng/ml and considered the presence or absence of an LH rise (≥12.6 IU/l) on the last day of monitoring before DYD initiation (i.e. the assays upper reference limit for follicular phase LH levels).

### DYD and DHD measurement

The concentration of dydrogesterone and dihydrodydrogesterone (DHD) was determined in 50 μl plasma samples using the high-performance liquid chromatography coupled with tandem mass spectrometry (HPLC/MS/MS) method, which had been previously validated and adjusted to the concentration ranges of 0.050–10.000 ng/ml for DYD and 0.500–100.000 ng/ml for DHD in plasma. Dipotassium ethylenediaminetetraacetic acid (K2EDTA) was used as an anticoagulant. The standard calibration curves covered the ranges of 0.050–10.000 ng/ml for DYD and 0.500–100.000 ng/ml for DHD. Plasma samples were precipitated with a solution of internal standards dDYD and dDHD (dextrorotatory form of the molecule) in 80% Acetonitrile (ACN). The supernatant was analysed using by HPLC/MS/MS by company QUINTA-ANALYTICA (Prague, Czech Republic).

### HPLC/MS/MS validation

The HPLC/MS/MS method used for the present samples was validated using historical controls, i.e. samples originally collected by Neumann *et al.* (2022), that had been kept stored at -80°C and that had previously been analysed on a different HPLC/MS/MS system. Validation was conducted at low, medium, and high DYD concentrations, with 10 samples for each concentration level. To assess the agreement between the two HPLC/MS/MS platforms, Bland-Altman plots were generated for both DYD and DHD (Bland and Altman, 1986).

### Estradiol and progesterone measurement

Estradiol in serum was measured using the Alinity i Estradiol assay (Abbott Laboratories. Alinity i Estradiol Reagent Kit, Abbott GmbH, Wiesbaden, Germany). Intra-assay coefficient of variation (CV) values ranged from 2.2% to 7.2% across low, medium, and high control levels. Inter-assay CVs ranged from 2.6% to 7.7%. The assay’s limit of detection (LoD), and limit of quantitation (LoQ) were 20 and 24 pg/ml, respectively. Progesterone in serum was measured using the ARCHITECT Progesterone assay (Abbott Laboratories. ARCHITECT Progesterone, Abbott GmbH, Wiesbaden, Germany), demonstrating intra-assay CVs ranging from 1.5% to 5.5%, and inter-assay CVs ≤6.2% for low controls, ≤2.9% for medium controls, and ≤3.9% for high controls. The LoD for progesterone was ∼0.1 ng/ml. The assays reliably measure up to 40 ng/ml for progesterone and 1000 pg/ml for estradiol (Reliable maximal values).

### Sample size

During the study period, we aimed to recruit 700 patients across four centres, with an estimated 500 patients undergoing FET in a PO-FET regimen. In a previous study, a difference of -22% in the ongoing pregnancy/live birth rate was observed in women undergoing HRT FET with 10 mg oral DYD (tid) monotherapy, comparing those below and above the 25th percentile of DYD concentrations in plasma (Neumann *et al.*, 2022). With 500 observations, the study had 80% power to detect a difference of −11.55% in live birth rate, potentially associated with DYD levels within the PO-FET protocol, assuming a 25% live birth rate above the 25th percentile.

### Statistical analysis

All analyses were primarily descriptive and exploratory. Descriptive statistics, including mean, standard deviation (SD), confidence interval (CI), or median, quartiles and range, as well as absolute numbers and relative proportions, were used to characterize patient demographics and outcomes. For hormonal values, observations above or below limits of quantification were set to these, and when empirical distribution functions were fitted to confirm the anticipated lognormal distributions, these were treated as censored observations. Concentration data were log-transformed before further analysis. For E2 and P levels, the patient sample was divided into below and above the 25th percentile (lower quartile) of concentrations at embryo transfer. DYD and DHD distributions were compared with historical data, and after confirming sufficient assay agreement by Bland-Altman analysis, previously established thresholds were used to stratify the population (specifically 0.71 ng/ml for DYD and 20.67 ng/ml for DHD for the lower quarter vs the rest). Implantation, pregnancy rates, and live birth rates were described by the risk difference with 95% confidence intervals between hormone quarters. This was stratified by day of embryo transfer (days 2-3 vs 4-5) before computing *P*-values. A smooth spline function was used to model the probability of live birth with confidence intervals separately as a function (of the logarithm) of DYD, DHD, P, and E2 levels. The odds ratio for live birth was estimated using logistic regression with an interaction term. Live birth rates were colour-coded in a scatter plot by hormone level quarters on the day of FET to explore potential combined effects like synergy, substitution, or interaction. A *P*-value <0.05 was considered statistically significant when verbalizing empirical findings with no adjustment for multiplicity of these exploratory statistics. All statistical analyses were performed using the software Jamovi (Version 2.3.28, Sydney, Australia) (https://www.jamovi.org) and R 4.4.0 (The R Foundation for Statistical Computing, Vienna, Austria).

## Results

### Patient flow and demographics

Between February 2021 and August 2023, 728 patients were included across four centres; of these, 559 underwent an embryo transfer in a PO-FET protocol, with plasma and serum samples collected, and complete outcome data available. Supplementary Fig. S1 shows the patient inclusion and flow. Supplementary Table S1 shows patient demographics. In summary, patients were on average 34 years old and weighed 70 kg; 92% were Caucasian; 73% were regularly cycling; and on average had a follicle size of 17.2 mm (±2.2), endometrial thickness of 9.2 mm (±2.1), and LH levels of 19.2 IU/l (±20.2) on day of last monitoring before DYD initiation. FET was performed on Day 2, 3, 4, or 5 in 4.6%, 12.7%, 3.4%, and 79.2% of cases, respectively; single frozen embryo transfer (sFET) was performed in 86% of cases. Clinical pregnancy and live birth rates per women undergoing FET were 32.6% (95% CI 28.73–36.51) and 27.1% (95% CI 23.37–30.75), respectively.

### Endocrine profile on day of FET

**Figure 2.**
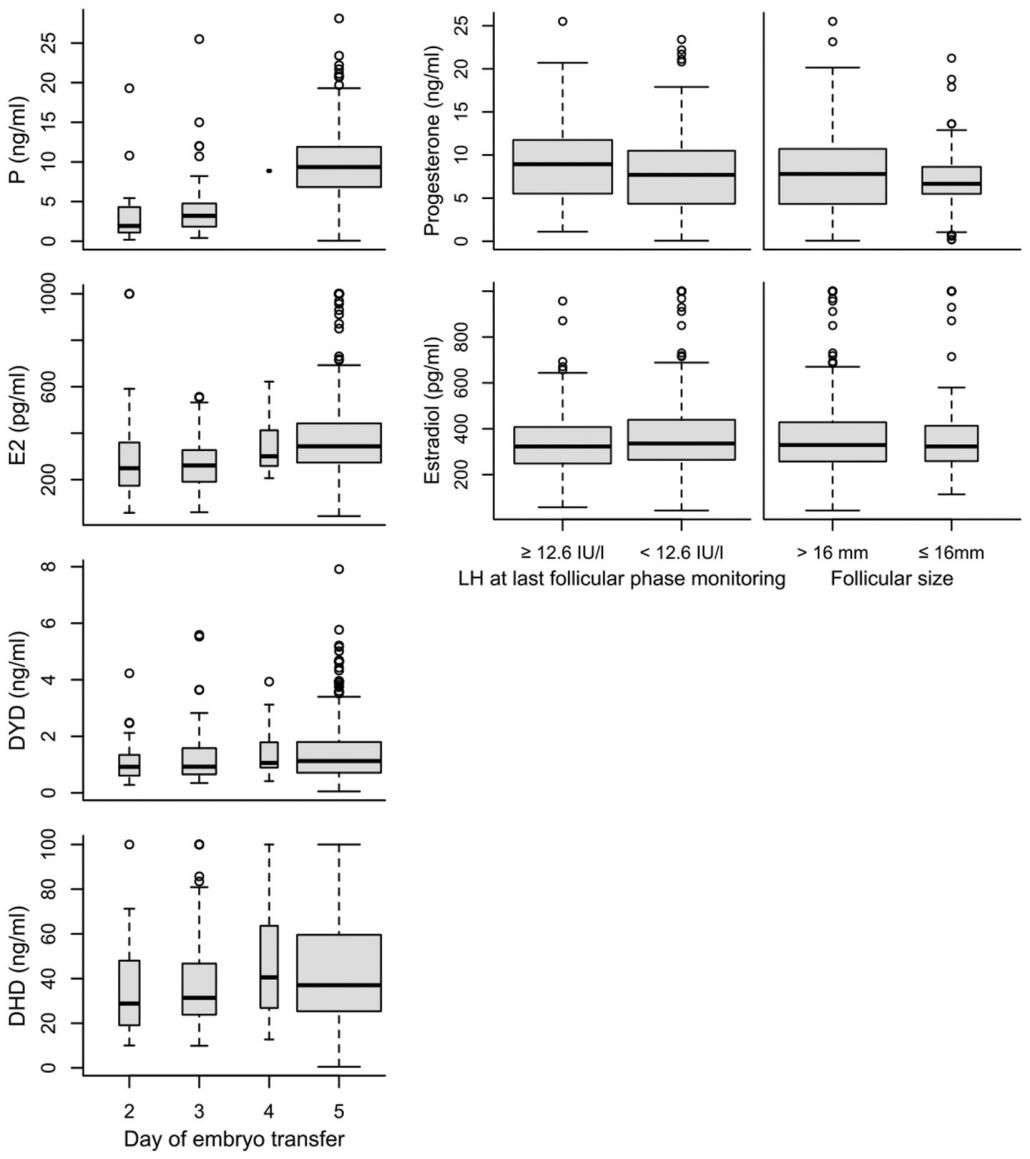
**Distribution of hormone values by day of FET.** Boxplots display serum hormone concentrations according to FET day (i.e. by increasing duration of dydrogesterone [DYD] intake and thus cycle progression), serum LH level, and lead follicular diameter at last monitoring before the initiation of DYD. FET was performed on Day 2 (n=26), Day 3 (n=71), Day 4 (n=19), and Day 5 (n=443) of DYD intake. Outliers were truncated at the upper limit of reliable quantification for DYD (n=1), 20α-dihydrodydrogesterone (DHD; n=24), progesterone (P; n=2), and estradiol (E2; n=12). FET, frozen–thawed embryo transfer; DYD, dydrogesterone; DHD, 20α-dihydrodydrogesterone; P, progesterone; E2, estradiol; IU/l, international units per litre; mm, millimeter.

Empirical distributions of DYD, DHD, P, and E2 with lognormal distribution functions overlaid are shown in Supplementary Fig. S2. Progesterone serum levels were strongly associated with cycle progression (ratio of geometric means 2.88, 95% CI 2.48–3.35; Fig. 2), while P and E2 levels on the day of FET showed little or no association with the initiation of the LH surge or with follicular size at the last monitoring in the follicular phase (Fig. 2; Supplementary Table S2). Supplementary Fig. S3 illustrates positive, albeit very weak relationships between individual serum progesterone levels on the day of FET with follicular size, LH levels, and E2 levels at last monitoring. Joint distributions of LH levels and follicular sizes of patients with and without P-levels indicative of ovulation had a large overlap.

**Figure 3.**
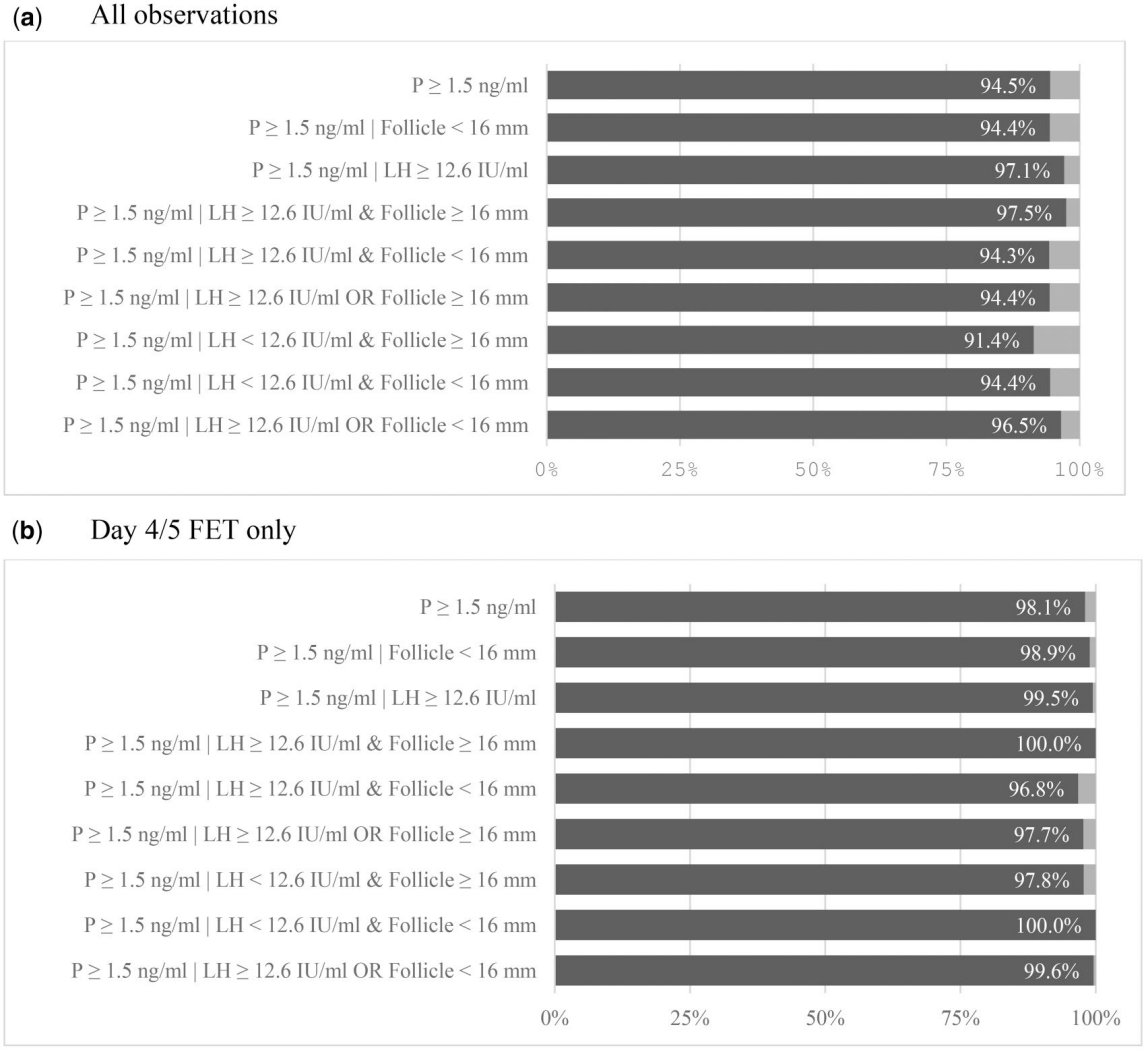
**Incidence of luteal progesterone elevation stratified by LH rise and follicle size.** Incidence of progesterone elevation indicative of ovulation (≥1.5 ng/ml) in women undergoing a dydrogesterone-based programmed-ovulatory FET protocol. (**a**) All luteal phase observations (n=558), stratified by LH level (≥12.6 or <12.6 IU/ml) and lead follicle diameter (≥16 or <16 mm) at the last monitoring before dydrogesterone initiation. (**b**) Subgroup of patients undergoing FET on Day 4 or 5 (n=462), with the same stratification. Percentages indicate the proportion of patients with progesterone ≥1.5 ng/ml. FET, frozen–thawed embryo transfer; P, progesterone; IU/l, international units per litre; mm, millimeter.

Progesterone serum levels indicative of ovulation (≥1.5 ng/ml) were observed in 98.1% of cases in the subgroup of patients undergoing FET on day 4/5 and in 94.5% of the total cohort (Fig. 3). Applying a higher threshold of >3.0 ng/ml, these proportions were 95.4% and 87.6%, respectively (data not shown in the figure). The incidence of progesterone elevation was higher in patients with LH ≥12.6 IU/ml but not in those with a lead follicle size <16 mm; combining both criteria or applying either/or conditions did not materially change the results (Fig. 3).

Given the apparent influence of embryo stage (i.e. cycle progression), further analysis focused on the FET day 4/5 subgroup. Of 13 patients with progesterone levels below 1.5 ng/ml (or 3.0 ng/ml) on the day of FET, five had levels above 3.0 ng/ml at the time of serum hCG measurement; four of these achieved clinical pregnancies and were counted towards the total with ovulatory-range progesterone levels. Among patients with a follicle size ≥16 mm at the last monitoring, 45.4% had LH ≥12.6 IU/l and 54.6% had LH <12.6 IU/l. Progesterone exceeded 1.5 and 3.0 ng/ml in 99.5% and 97.9% of patients with LH ≥12.6 IU/l, respectively, and in 97.8% and 95.0% of those with LH <12.6 IU/l; when the lead follicle diameter was <16 mm at last monitoring before DYD initiation, no progesterone values in the anovulatory range (<1.5 ng/ml) were observed.

DYD and DHD concentration distributions on Days 3–6 of intake were consistent with previous findings (Neumann *et al.*, 2022) (Supplementary Table S3). While DYD and DHD were highly correlated (r_DYD,DHD_ = 0.95), P-levels were very weakly correlated with either (r_DYD,P_ = 0.02, r_DHD,P_ = 0.03) or with E2 levels (r_P,E_ = 0.21) using rank correlation.

### HPLC/MS/MS method validation

In Bland–Altmann analysis, for DYD, the bias was 10.03% (95% CI 7.74–12.37) with limits of agreement ranging from −1.28% to 22.62%. For DHD, the bias was −6.7% (95% CI −8.96 to −4.39), with limits of agreement from −17.75% to 5.84%. Visual inspections of the plots (Supplementary Fig. S4) indicated good method agreement across the range of concentrations. Accordingly, previously established thresholds for DYD and DHD (i.e. 0.71 ng/ml for DYD & 20.67 ng/ml for DHD) were applied to dichotomize the present population into low versus normal-high concentrations (Neumann *et al.*, 2022) for these two analytes.

### Treatment outcome by hormonal levels on day of FET

**Figure 4.**
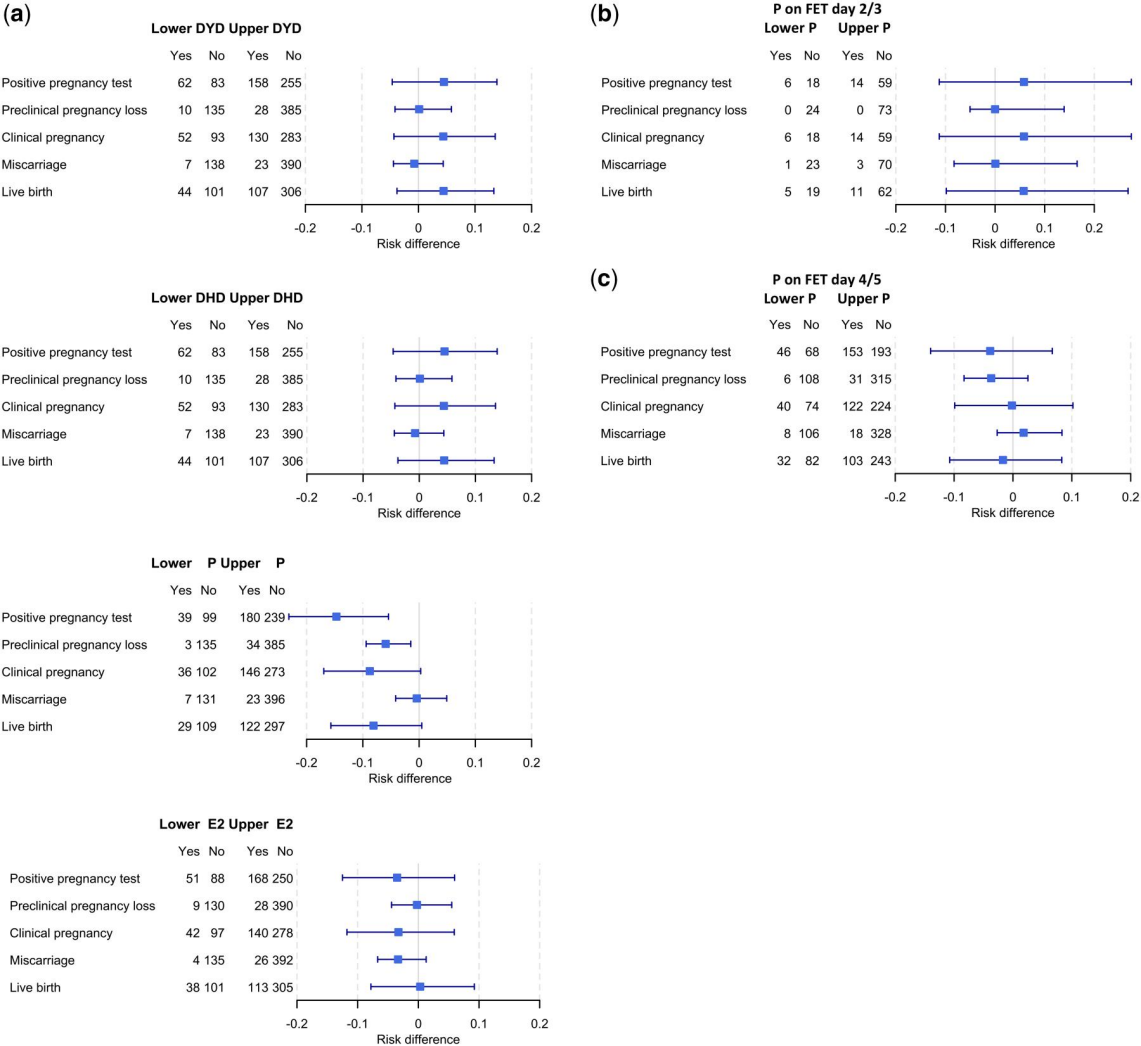
**Treatment outcomes by hormonal levels on the day of FET.** (**a**) Risk differences with confidence intervals for treatment outcome from implantation to live birth calculated per patient undergoing frozen–thawed embryo transfer (FET), in subgroups of low versus normal–high hormone levels on the day of FET. (**b**) Risk differences in subgroups of low versus normal–high progesterone levels in patients undergoing FET on Day 2 or 3 of embryonic development (2/3). (**c**) Risk differences in subgroups of low versus normal–high progesterone levels in patients undergoing FET on Day 4 or 5 of embryonic development (4/5). DYD, dydrogesterone; DHD, 20α-dihydrodydrogesterone; P, progesterone; E2, estradiol; FET, frozen–thawed embryo transfer.

**Figure 5.**
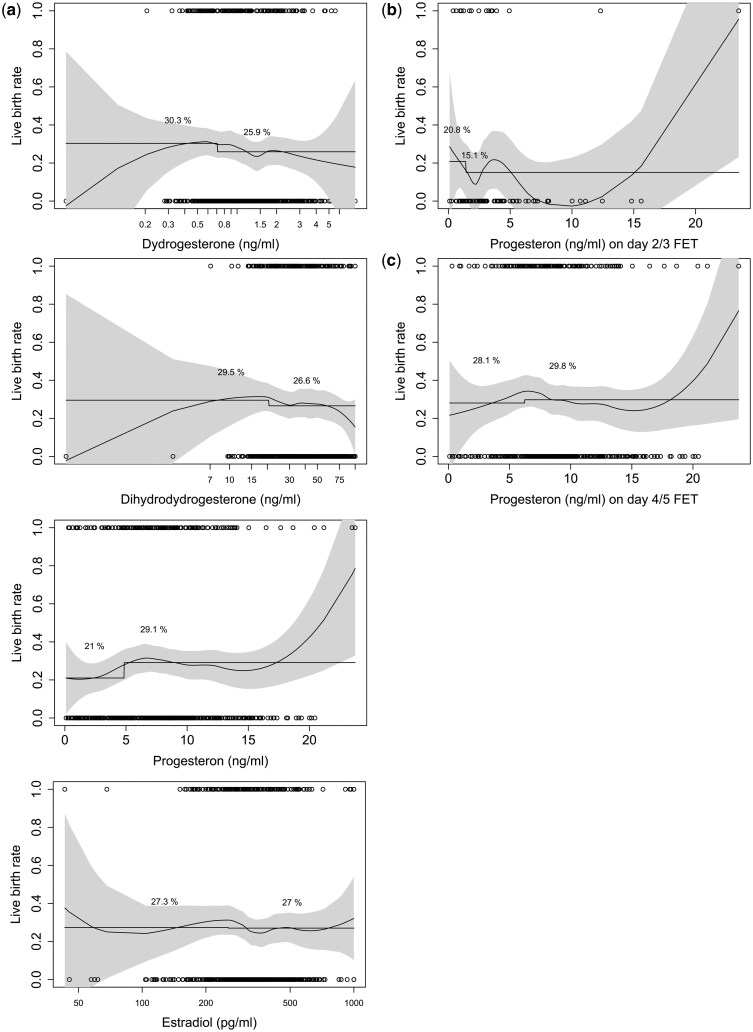
**Live birth probability across hormone levels with FET day-stratified progesterone analysis.** (**a**) Probability of live birth as a function of logarithm of dydrogesterone, 20α-dihydrodydrogesterone, progesterone (P), and estradiol concentrations, observations: smooth spline with confidence band and dichotomization at the lower quartile. (**b **and** c**) Probability of live birth as a function of logarithm of P observations: smooth spline with confidence band and dichotomization at the lower quartile, presented separately for day of FET. FET, frozen-thawed embryo transfer.

**Fig. 6.**
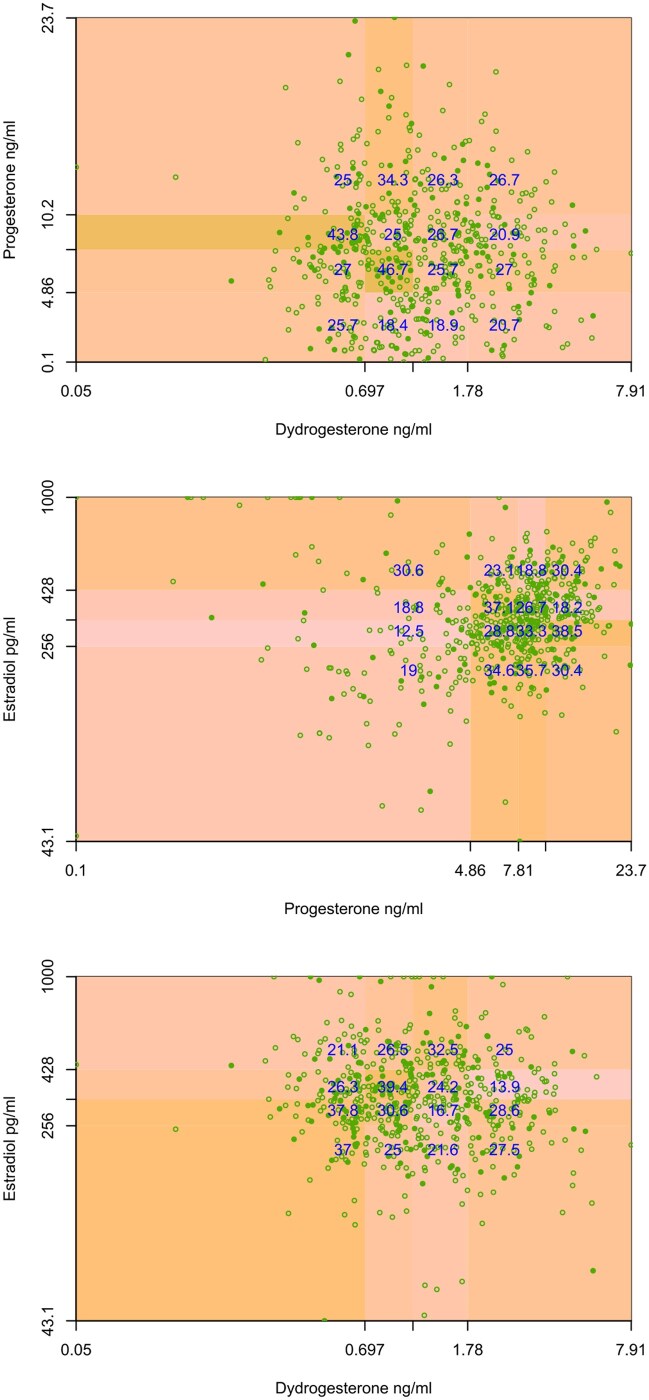
**Predicted live birth by hormone level combinations on day of FET.** Scatterplots stratified for logarithm of hormone quarters for combinations of hormone levels measured on the day of FET. Open circles represent cycles without a live birth, filled circles represent cycles resulting in a live birth. The background shading indicates the estimated probability of live birth, with darker shades denoting higher probabilities. Numbers in blue indicate live birth rate. ng/ml, nanogram per millilitre; FET, frozen–thawed embryo transfer.

Figure 4a illustrates the risk differences with confidence intervals for implantation, clinical pregnancy, early and late pregnancy loss, and live birth in FET patients, categorized into subgroups with low versus normal–high hormone levels. Patients with low progesterone had a lower positive pregnancy test rate and a lower preclinical pregnancy loss rate. When stratified by FET timing (Day 2/3 vs 4/5, shown in Fig. 4b and c), the risk differences for live birth were marginal and not statistically significant between low and normal–high hormone values (DYD RD −5.3%, 95% CI −14.0 to 3.2, *P* = 0.227; DHD RD −4.0%, 95% CI −14.6 to 6.0, *P* = 0.428; Progesterone RD 2.9%, 95% CI −7.0 to 12.3, *P* = 0.597; Estradiol RD −3.3%, 95% CI −12.2 to 5.3, *P* = 0.487). Logistic regressions from live birth on hormone levels at FET resulted in 95%-confidence limits for the odds ratio (Supplementary Table S4) on different sides of 1, even when a two-way interaction was considered. Figure 5a further depicts these findings for live birth outcomes using a spline curve across the full range of individual hormone concentrations, with separate analyses for progesterone stratified by FET timing (Fig. 5b and c). Figure 6 demonstrates the absence of interaction effects on live birth rates between relevant hormone pairs at both the low and high ends of the concentration range using a scatter plot overlaid with a colour-coded probability gradient indicating the likelihood of live birth across hormone concentrations.

## Discussion

In this study, we demonstrate that oral DYD 10 mg (tid), administered to induce receptivity and schedule FET in the late follicular phase, is compatible with progesterone levels indicative of ovulation in the vast majority of cases, largely independent of an LH surge and follicular size at the time of DYD initiation. Although the protocol defined ≥16 mm follicular diameter as the target threshold for DYD initiation, in practice, a small proportion of patients began treatment at smaller sizes, for example when LH surges occurred. In a small proportion of cases, a leading follicle could not be visualized by sonography, yet endometrial thickness and serum estradiol levels were considered sufficient. This reflects the variability inherent in routine clinical monitoring and decision-making in natural follicular phase FET cycles, where treatment has to be individualized based on a combination of follicular size, hormonal dynamics, and practical factors. Importantly, such variability did not appear to systematically affect the primary finding which is that a low progesterone level on the day of FET, indicative of late or absent ovulation, or low corpus luteum activity, is not associated with the chances of a live birth. Similarly, no associations were observed between DYD, DHD, or E2 levels on the day of FET and live birth rates, nor were there interactions between the studied hormones affecting outcomes. These findings suggest that oral DYD can effectively replace an hCG injection to schedule FET in ovulatory women. The use of hCG in an NC is typically referred to as a ‘managed NC’. For the novel approach presented here, we propose the term ‘Programmed-Ovulatory FET cycle’, highlighting that the window of implantation (WOI) is ‘programmed’ by DYD initiation, similar to a ‘programmed’ HRT cycle, while ovulation may still occur naturally.

A potential benefit of the PO-FET protocol demonstrated in this study is that hormone measurements on the day of FET may be unnecessary. This contrasts with HRT FET protocols using only vaginal progesterone, where hormone measurements and ‘rescue’ interventions, such as adding another progestin to LPS, are recommended by several authors for women who have been identified with low P levels (Labarta *et al.*, 2021; du Boulet *et al.*, 2022; Mackens *et al.*, 2023; Metello *et al.*, 2024). Notably, in the unadjusted analysis presented here, a low serum progesterone level on the day of FET was associated with lower rates of positive hCG tests. However, 17.4% of patients underwent FET with embryos on Days 2 or 3 of extra-corporal development, which is linked to a poorer prognosis, as shown in Fig. 5. Earlier embryo transfers, however, also correlate with lower progesterone levels on the day of FET as shown in Fig. 3, because these patients were less advanced in their cycles and had not yet reached an ovulatory or post-ovulatory state. After adjusting for the day of embryo transfer, low progesterone levels were no longer associated with live birth outcomes.

In a previous study (Neumann *et al.*, 2022), we demonstrated that a monotherapy of daily oral 10 mg DYD (tid) in a confirmed anovulatory HRT FET protocol was associated with a live birth rate of 28% in women with serum DYD levels above the 25th percentile, compared to 6% in those below. In the present study, we confirm the pharmacokinetic data for DYD and DHD from the previous study and demonstrate that DYD/DHD levels below the 25th percentile are not associated with outcomes in women who predominantly ovulate and who thus have endogenous progesterone production, likely an effect of the dual progestogenic support in this protocol (i.e. endogenous and exogenous progestogenic activity). It can be argued that, even if a woman remains anovulatory in the PO-FET protocol, oral DYD alone should suffice to establish and maintain pregnancy in most cases.

A substantial body of literature suggests that anovulatory FET cycles may be associated with worse maternal and fetal outcomes (von Versen-Höynck *et al.*, 2019; Moreno-Sepulveda *et al.*, 2021; Rosalik *et al.*, 2021; Conrad *et al.*, 2022; von Versen-Höynck and Griesinger, 2022). Accordingly, it seems prudent to develop and routinely use FET protocols that allow or support ovulation. Recently, the hypothesis has gained renewed attention that the LH surge is preceded by a progesterone surge (Dozortsev and Diamond, 2020). Notably, in the present study, progesterone levels and ovulation appeared to be only weakly associated to the onset of LH elevation at the final monitoring. This finding raises the intriguing possibility that oral DYD may have an LH surge-inducing effect, a hypothesis warranting further investigation.

In this study, we were unable to detect progesterone levels indicative of ovulation in 2%–5% of patients. This could reflect a detection error (e.g. insufficiently frequent longitudinal sampling) or true anovulation. If a small subset of women indeed remained anovulatory under the PO-FET protocol, it may represent a natural phenomenon of low-frequency cycle instability in an infertile population. Alternatively, it could be speculated that oral DYD modulated pituitary LH release via negative feedback in women susceptible to this effect. Supporting this, a recent phase I study demonstrated that continuous oral administration of 10 mg DYD (tid) from the start of the menstrual cycle rendered ∼70% of normally cycling, healthy women anovulatory over multiple cycles (Griesinger *et al.*, 2024). This finding underscores that even progestins considered to have minimal interference with the pituitary gland, such as low-dose dydrogesterone (summarized in Griesinger *et al.*, 2019), may negatively affect ovulation, corpus luteum formation, and maintenance. It is therefore critical to scrutinize LPS practices in mono-ovulatory FET cycles for potential adverse effects on corpus luteum activity and maintenance at the transition into early pregnancy (i.e. higher doses of exogenous progesterone may suppress the endogenous corpus luteum activity at a critical moment in time when early pregnancy ensues). In the PO-FET protocol using oral DYD, the absence of ovulation could be conveniently monitored through progesterone measurement in serum, alternatively in saliva or urine prior to embryo transfer (Cekan *et al.*, 1986; Ellison, 1993; Li *et al.*, 2002; Schiffer *et al.*, 2019). For women with confirmed anovulation, it remains to be determined whether hCG administration as a rescue in the PO-FET could induce the formation of a fully functional corpus luteum.

A similar approach to the PO-FET protocol has recently been introduced, utilizing vaginal micronized progesterone in what is termed a Progesterone-modified NC (P4mNC) (Kornilov *et al.*, 2024) or the Natural Proliferative Phase (NPP) protocol (Mendes Godinho *et al.*, 2024). In the P4mNC protocol, vaginal administration of 200 mg progesterone twice daily begins when the dominant follicle exceeds 16 mm in size and the endometrial lining is at least 7 mm thick. In the NPP protocol, vaginal progesterone is initiated during the proliferative phase as soon as the endometrium reaches a thickness of at least 7 mm and ovulation is ruled out, regardless of the dominant follicle size. In both protocols, ovulation, and the interaction of vaginally administered bio-identical progesterone with corpus luteum activity cannot be hormonally monitored. Additionally, it remains unclear whether the potential interactive effect of exogenous sex-steroid administration with ovulation is truly independent of the timing of initiation relative to follicular size and progression towards mid-cycle as suggested for the NPP protocol. Accordingly, these approaches need further study.

Generally, there is controversy in the literature, about whether LPS with a progestin may benefit outcomes of NC or managed NC FET (Mizrachi *et al.*, 2021; Jiang *et al.*, 2022, 2023; Alsbjerg *et al.*, 2024; Saupstad *et al.*, 2024a). It is also unclear whether progesterone levels in the luteal phase of NC or managed NCs reliably predict pregnancy (Gaggiotti-Marre *et al.*, 2020; Lawrenz *et al.*, 2023a; Saupstad *et al.*, 2024b). Moreover, it remains unknown whether low progesterone levels in NCs are more common in women of advanced age (Pfister *et al.*, 2019), exercising women (De Souza *et al.*, 2010), or women with distinct eating patterns (Andrews *et al.*, 2015), potentially limiting such associations to subgroups. The PO-FET protocol presented here is designed to combine the benefits of scheduling FET with the inclusion of LPS. However, its potential efficacy advantage over NC or managed NC protocols remains to be determined, however.

Finally, for any FET protocol involving pharmaceutical interventions (e.g. ovarian stimulation, hCG administration, or sex-steroid supplementation), luteal phase, and early pregnancy characteristics must be thoroughly understood, and the efficacy as well as the (maternal and fetal) safety should be evaluated in large-scale pragmatic studies. The PO-FET protocol presented here enables scheduling of the implantation window, supports ovulation and corpus luteum formation, provides dual gestagenic support, and may eliminate the need for sex-steroid measurements on the day of FET. It facilitates monitoring of corpus luteum activity during the luteal phase and early pregnancy, is injection-free, and has low drug costs. This protocol needs to be tested in RCTs against other ovulatory FET protocols, such as the NC FET or managed NC FET.

## Supplementary Material

hoaf058_Supplementary_Data

## Data Availability

The analysis dataset and statistical code underlying this study are currently being curated and will be made publicly available on Zenodo and GitHub under the dataset title ‘PO_FET_Luebeck’. A DOI-linked Zenodo repository will be established to ensure long-term accessibility and transparency. In the meantime, the data are available from the corresponding author upon reasonable request.

## References

[hoaf058-B1] Al-Hasani S , Ozmen B, Koutlaki N, Schoepper B, Diedrich K, Schultze-Mosgau A. Three years of routine vitrification of human zygotes: is it still fair to advocate slow-rate freezing? Reprod Biomed Online 2007;14:288–293. 17359578 10.1016/s1472-6483(10)60869-3

[hoaf058-B2] Alsbjerg B , Kesmodel US, Humaidan P, Bungum L. The natural menstrual cycle revisited—can natural cycle be trusted. J Ovarian Res 2024;17:153. 39039530 10.1186/s13048-024-01469-2 PMC11265377

[hoaf058-B3] Andrews MA , Schliep KC, Wactawski-Wende J, Stanford JB, Zarek SM, Radin RG, Sjaarda LA, Perkins NJ, Kalwerisky RA, Hammoud AO et al Dietary factors and luteal phase deficiency in healthy eumenorrheic women. Hum Reprod 2015;30:1942–1951. 26082480 10.1093/humrep/dev133 PMC4507331

[hoaf058-B4] Asserhøj LL , Spangmose AL, Aaris Henningsen AK, Clausen TD, Ziebe S, Jensen RB, Pinborg A. Adverse obstetric and perinatal outcomes in 1,136 singleton pregnancies conceived after programmed frozen embryo transfer (FET) compared with natural cycle FET. Fertil Steril 2021;115:947–956. 33461756 10.1016/j.fertnstert.2020.10.039

[hoaf058-B5] Bland JM , Altman DG. Statistical methods for assessing agreement between two methods of clinical measurement. Lancet 1986;1:307–310. 2868172

[hoaf058-B6] Busnelli A , Schirripa I, Fedele F, Bulfoni A, Levi-Setti PE. Obstetric and perinatal outcomes following programmed compared to natural frozen-thawed embryo transfer cycles: a systematic review and meta-analysis. Hum Reprod 2022;37:1619–1641. 35553678 10.1093/humrep/deac073

[hoaf058-B7] Cédrin-Durnerin I , Isnard T, Mahdjoub S, Sonigo C, Seroka A, Comtet M, Herbemont C, Sifer C, Grynberg M. Serum progesterone concentration and live birth rate in frozen-thawed embryo transfers with hormonally prepared endometrium. Reprod Biomed Online 2019;38:472–480. 30642638 10.1016/j.rbmo.2018.11.026

[hoaf058-B8] Cekan SZ , Beksac MS, Wang E, Shi S, Masironi B, Landgren BM, Diczfalusy E. The prediction and/or detection of ovulation by means of urinary steroid assays. Contraception 1986;33:327–345. 3731775 10.1016/0010-7824(86)90095-8

[hoaf058-B9] Conrad KP , Petersen JW, Chi YY, Zhai X, Li M, Chiu KH, Liu J, Lingis MD, Williams RS, Rhoton-Vlasak A et al Maternal cardiovascular dysregulation during early pregnancy after in vitro fertilization cycles in the absence of a corpus luteum. Hypertension 2019;74:705–715. 31352818 10.1161/HYPERTENSIONAHA.119.13015 PMC6687559

[hoaf058-B10] Conrad KP , von Versen-Höynck F, Baker VL. Potential role of the corpus luteum in maternal cardiovascular adaptation to pregnancy and preeclampsia risk. Am J Obstet Gynecol 2022;226:683–699. 34437863 10.1016/j.ajog.2021.08.018

[hoaf058-B11] De Souza MJ , Toombs RJ, Scheid JL, O’Donnell E, West SL, Williams NI. High prevalence of subtle and severe menstrual disturbances in exercising women: confirmation using daily hormone measures. Hum Reprod 2010;25:491–503. 19945961 10.1093/humrep/dep411

[hoaf058-B12] Depenbusch M, Graspeuntner S, Lupatsii M, Masuch A, Hamala N, Pfeffer I, Schultze-Mosgau A, Eggersmann T, Griesinger G. O-276 Implantation failure is not associated with seven different vaginal bacterial community-state types; however, a distinct signature, including Ureaplasma parvum, is strongly linked to treatment failure. *Hum Reprod* 2024;39:deae108.323.

[hoaf058-B13] Dozortsev DI , Diamond MP. Luteinizing hormone-independent rise of progesterone as the physiological trigger of the ovulatory gonadotropins surge in the human. Fertil Steril 2020;114:191–199. 32741458 10.1016/j.fertnstert.2020.06.016

[hoaf058-B14] Du Boulet B , Ranisavljevic N, Mollevi C, Bringer-Deutsch S, Brouillet S, Anahory T. Individualized luteal phase support based on serum progesterone levels in frozen-thawed embryo transfer cycles maximizes reproductive outcomes in a cohort undergoing preimplantation genetic testing. Front Endocrinol (Lausanne) 2022;13:1051857. 36531476 10.3389/fendo.2022.1051857 PMC9755854

[hoaf058-B15] Eggersmann TK , Wolthuis A, van Amsterdam PH, Griesinger G. Lack of analytical interference of dydrogesterone in progesterone immunoassays. Clin Chem Lab Med 2022;60:1039–1045. 35535412 10.1515/cclm-2022-0174

[hoaf058-B16] Ellison PT. Measurements of salivary progesterone. Ann N Y Acad Sci 1993;694:161–176. 8215052 10.1111/j.1749-6632.1993.tb18350.x

[hoaf058-B17] Fatemi HM , Kyrou D, Bourgain C, Van den Abbeel E, Griesinger G, Devroey P. Cryopreserved-thawed human embryo transfer: spontaneous natural cycle is superior to human chorionic gonadotropin-induced natural cycle. Fertil Steril 2010;94:2054–2058. 20097333 10.1016/j.fertnstert.2009.11.036

[hoaf058-B18] Gaggiotti-Marre S , Álvarez M, González-Foruria I, Parriego M, Garcia S, Martínez F, Barri PN, Polyzos NP, Coroleu B. Low progesterone levels on the day before natural cycle frozen embryo transfer are negatively associated with live birth rates. Hum Reprod 2020;35:1623–1629. 32478389 10.1093/humrep/deaa092

[hoaf058-B19] Ghobara T , Gelbaya TA, Ayeleke RO. Cycle regimens for frozen-thawed embryo transfer. Cochrane Database Syst Rev 2017;6:CD003414. 10.1002/14651858.CD003414.pub3 PMC6483463 28675921

[hoaf058-B20] Ginström Ernstad E , Wennerholm UB, Khatibi A, Petzold M, Bergh C. Neonatal and maternal outcome after frozen embryo transfer: increased risks in programmed cycles. Am J Obstet Gynecol 2019;221:126.e1–126.e18. 10.1016/j.ajog.2019.03.010 30910545

[hoaf058-B21] Griesinger G , Blockeel C, Kahler E, Pexman-Fieth C, Olofsson JI, Driessen S, Tournaye H. Dydrogesterone as an oral alternative to vaginal progesterone for IVF luteal phase support: a systematic review and individual participant data meta-analysis. PLoS One 2020;15:e0241044. 33147288 10.1371/journal.pone.0241044 PMC7641447

[hoaf058-B22] Griesinger G , Blockeel C, Sukhikh GT, Patki A, Dhorepatil B, Yang DZ, Chen ZJ, Kahler E, Pexman-Fieth C, Tournaye H. Oral dydrogesterone versus intravaginal micronized progesterone gel for luteal phase support in IVF: a randomized clinical trial. Hum Reprod 2018;33:2212–2221. 30304457 10.1093/humrep/dey306 PMC6238366

[hoaf058-B23] Griesinger G , De, sai S, Yadav L. P-642 The effect of oral dydrogesterone (DYD) on the hypothalamic-pituitary-ovarian (HPO) axis (gonadotropin and sex-steroid levels) and ovulation inhibition: a double-blind, phase I, randomized-controlled, parallel-group trial. Hum Reprod 2024;39:deae108.974. 10.1093/humrep/deae108.974

[hoaf058-B24] Griesinger G , Tournaye H, Macklon N, Petraglia F, Arck P, Blockeel C, van Amsterdam P, Pexman-Fieth C, Fauser BC. Dydrogesterone: pharmacological profile and mechanism of action as luteal phase support in assisted reproduction. Reprod Biomed Online 2019;38:249–259. 30595525 10.1016/j.rbmo.2018.11.017

[hoaf058-B25] Groenewoud ER , Cohlen BJ, Al-Oraiby A, Brinkhuis EA, Broekmans FJ, de Bruin JP, van den Dool G, Fleisher K, Friederich J, Goddijn M et al A randomized controlled, non-inferiority trial of modified natural versus artificial cycle for cryo-thawed embryo transfer. Hum Reprod 2016;31:1483–1492. 27179265 10.1093/humrep/dew120 PMC5853593

[hoaf058-B26] Groenewoud ER , Kollen BJ, Macklon NS, Cohlen BJ. Spontaneous LH surges prior to HCG administration in unstimulated-cycle frozen-thawed embryo transfer do not influence pregnancy rates. Reprod Biomed Online 2012;24:191–196. 22197128 10.1016/j.rbmo.2011.11.003

[hoaf058-B27] Ho VNA , Pham TD, Nguyen NT, Wang R, Norman RJ, Mol BW, Ho TM, Vuong LN. Livebirth rate after one frozen embryo transfer in ovulatory women starting with natural, modified natural, or artificial endometrial preparation in Viet Nam: an open-label randomised controlled trial. Lancet 2024;404:266–275. 38944045 10.1016/S0140-6736(24)00756-6

[hoaf058-B28] Hu KL , Zhang D, Li R. Endometrium preparation and perinatal outcomes in women undergoing single-blastocyst transfer in frozen cycles. Fertil Steril 2021;115:1487–1494. 33487443 10.1016/j.fertnstert.2020.12.016

[hoaf058-B29] International Federation of Fertility Societies’ Surveillance (IFFS). 2022: Global Trends in Reproductive Policy and Practice, 9th Edition. Glob Reprod Health 2022;7:e58.

[hoaf058-B30] Jiang WJ , Sun ZG, Song JY. Impact of progesterone-free luteal phase support following natural cycle frozen embryo transfer: study protocol for a multicenter, non-inferiority, randomized controlled trial. Front Med (Lausanne) 2022;9:1014946. 36457576 10.3389/fmed.2022.1014946 PMC9705326

[hoaf058-B31] Jiang Y , Wang L, Shen H, Wang B, Wu J, Hu K, Wang Y, Ma B, Zhang X. The effect of progesterone supplementation for luteal phase support in natural cycle frozen embryo transfer: a systematic review and meta-analysis based on randomized controlled trials. Fertil Steril 2023;119:597–605. 36574915 10.1016/j.fertnstert.2022.12.035

[hoaf058-B32] Kornilov N , Polyakov A, Mungalova A, Yakovleva L, Yakovlev P. Progesterone-modified natural cycle preparation for frozen embryo transfer. Reprod Biomed Online 2024;49:104350. 39244908 10.1016/j.rbmo.2024.104350

[hoaf058-B33] Labarta E , Mariani G, Holtmann N, Celada P, Remohí J, Bosch E. Low serum progesterone on the day of embryo transfer is associated with a diminished ongoing pregnancy rate in oocyte donation cycles after artificial endometrial preparation: a prospective study. Hum Reprod 2017;32:2437–2442. 29040638 10.1093/humrep/dex316

[hoaf058-B34] Labarta E , Mariani G, Paolelli S, Rodriguez-Varela C, Vidal C, Giles J, Bellver J, Cruz F, Marzal A, Celada P et al Impact of low serum progesterone levels on the day of embryo transfer on pregnancy outcome: a prospective cohort study in artificial cycles with vaginal progesterone. Hum Reprod 2021;36:683–692. 33340402 10.1093/humrep/deaa322

[hoaf058-B35] Lawrenz B , Ata B, Kalafat E, Melado L, ElKhatib I, Del Gallego R, Fatemi H. Are systemic progesterone levels in true natural cycle euploid frozen embryo transfers with luteal phase support predictive for ongoing pregnancy rates? Hum Reprod 2023a;38:1318–1324. 37196321 10.1093/humrep/dead104

[hoaf058-B36] Lawrenz B , Melado L, Fatemi HM. Frozen embryo transfers in a natural cycle: how to do it right. Curr Opin Obstet Gynecol 2023b;35:224–229. 36924405 10.1097/GCO.0000000000000862

[hoaf058-B37] Li H , Chen J, Overstreet JW, Nakajima ST, Lasley BL. Urinary follicle-stimulating hormone peak as a biomarker for estimating the day of ovulation. Fertil Steril 2002;77:961–966. 12009351 10.1016/s0015-0282(02)02998-9

[hoaf058-B38] Litwicka K , Mencacci C, Arrivi C, Varricchio MT, Caragia A, Minasi MG, Greco E. HCG administration after endogenous LH rise negatively influences pregnancy rate in modified natural cycle for frozen-thawed euploid blastocyst transfer: a pilot study. J Assist Reprod Genet 2018;35:449–455. 29147846 10.1007/s10815-017-1089-x PMC5904062

[hoaf058-B39] Lüth T , Graspeuntner S, Neumann K, Kirchhoff L, Masuch A, Schaake S, Lupatsii M, Tse R, Griesinger G, Trinh J et al Improving analysis of the vaginal microbiota of women undergoing assisted reproduction using nanopore sequencing. J Assist Reprod Genet 2022;39:2659–2667. 36223010 10.1007/s10815-022-02628-4 PMC9722992

[hoaf058-B40] Lutjen P , Trounson A, Leeton J, Findlay J, Wood C, Renou P. The establishment and maintenance of pregnancy using in vitro fertilization and embryo donation in a patient with primary ovarian failure. Nature 1984;307:174–175. 6690997 10.1038/307174a0

[hoaf058-B41] Mackens S , Pais F, Drakopoulos P, Amghizar S, Roelens C, Van Landuyt L, Tournaye H, De Vos M, Blockeel C. Individualized luteal phase support using additional oral dydrogesterone in artificially prepared frozen embryo transfer cycles: is it beneficial? Reprod Biomed Online 2023;46:939–945. 37012101 10.1016/j.rbmo.2023.02.007

[hoaf058-B42] Mackens S , Stubbe A, Santos-Ribeiro S, Van Landuyt L, Racca A, Roelens C, Camus M, De Vos M, van de Vijver A, Tournaye H et al To trigger or not to trigger ovulation in a natural cycle for frozen embryo transfer: a randomized controlled trial. Hum Reprod 2020;35:1073–1081. 32395750 10.1093/humrep/deaa026

[hoaf058-B43] Magnusson Å , Hanevik HI, Laivuori H, Loft A, Piltonen T, Pinborg A, Bergh C. Endometrial preparation protocols prior to frozen embryo transfer—convenience or safety? Reprod Biomed Online 2024;48:103587. 37949762 10.1016/j.rbmo.2023.103587

[hoaf058-B44] Maignien C , Bourdon M, Marcellin L, Guibourdenche J, Chargui A, Patrat C, Plu-Bureau G, Chapron C, Santulli P. Clinical factors associated with low serum progesterone levels on the day of frozen blastocyst transfer in hormonal replacement therapy cycles. Hum Reprod 2022;37:2570–2577. 36125015 10.1093/humrep/deac199

[hoaf058-B45] Melo P , Chung Y, Pickering O, Price MJ, Fishel S, Khairy M, Kingsland C, Lowe P, Petsas G, Rajkhowa M et al Serum luteal phase progesterone in women undergoing frozen embryo transfer in assisted conception: a systematic review and meta-analysis. Fertil Steril 2021;116:1534–1556. 34384594 10.1016/j.fertnstert.2021.07.002

[hoaf058-B46] Mendes Godinho C , Soares SR, Nunes SG, Martínez JMM, Santos-Ribeiro S. Natural proliferative phase frozen embryo transfer-a new approach which may facilitate scheduling without hindering pregnancy outcomes. Hum Reprod 2024;39:1089–1097. 38531673 10.1093/humrep/deae061

[hoaf058-B47] Metello J , Tomás C, Ferreira P, Natário I, Santos-Ribeiro S. Impact of dydrogesterone use in cycles with low progesterone levels on the day of frozen embryo transfer. J Assist Reprod Genet 2024;41:1577–1584. 38676842 10.1007/s10815-024-03118-5 PMC11224062

[hoaf058-B48] Mizrachi Y , Horowitz E, Ganer Herman H, Farhi J, Raziel A, Weissman A. Should women receive luteal support following natural cycle frozen embryo transfer? A systematic review and meta-analysis. Hum Reprod Update 2021;27:643–650. 33829269 10.1093/humupd/dmab011

[hoaf058-B49] Moreno-Sepulveda J , Espinós JJ, Checa MA. Lower risk of adverse perinatal outcomes in natural versus artificial frozen-thawed embryo transfer cycles: a systematic review and meta-analysis. Reprod Biomed Online 2021;42:1131–1145. 33903031 10.1016/j.rbmo.2021.03.002

[hoaf058-B50] Neumann K , Depenbusch M, Schultze-Mosgau A, Griesinger G. Characterization of early pregnancy placental progesterone production by use of dydrogesterone in programmed frozen-thawed embryo transfer cycles. Reprod Biomed Online 2020a;40:743–751. 32336650 10.1016/j.rbmo.2020.01.019

[hoaf058-B51] Neumann K , Depenbusch M, Schultze-Mosgau A, Griesinger G. Strong variation in progesterone production of the placenta in early pregnancy—what are the clinical implications? Reprod Biomed Online 2020b;41:748–749. 32800444 10.1016/j.rbmo.2020.07.009

[hoaf058-B52] Neumann K , Masuch A, Vonthein R, Depenbusch M, Schultze-Mosgau A, Eggersmann TK, Griesinger G. Dydrogesterone and 20α-dihydrodydrogesterone plasma levels on day of embryo transfer and clinical outcome in an anovulatory programmed frozen-thawed embryo transfer cycle: a prospective cohort study. Hum Reprod 2022;37:1183–1193. 35323905 10.1093/humrep/deac045

[hoaf058-B53] Pfister A , Crawford NM, Steiner AZ. Association between diminished ovarian reserve and luteal phase deficiency. Fertil Steril 2019;112:378–386. 31056309 10.1016/j.fertnstert.2019.03.032

[hoaf058-B54] Roelens C , Racca A, Mackens S, Van Landuyt L, Buelinckx L, Gucciardo L, Tournaye H, De Vos M, Blockeel C. Artificially prepared vitrified-warmed embryo transfer cycles are associated with an increased risk of pre-eclampsia. Reprod Biomed Online 2022;44:915–922. 35282993 10.1016/j.rbmo.2021.12.004

[hoaf058-B55] Rosalik K , Carson S, Pilgrim J, Luizzi J, Levy G, Heitmann R, Pier B. Effects of different frozen embryo transfer regimens on abnormalities of fetal weight: a systematic review and meta-analysis. Hum Reprod Update 2021;28:1–14. 34865039 10.1093/humupd/dmab037

[hoaf058-B56] Saupstad M , Bergenheim S, Colombo C, Bogstad JW, Klajnbard A, Freiesleben NLC, Alsbjerg B, Oxlund-Mariegaard BS, Løkkegaard E, Husth M et al O-204 Optimal timing and endometrial preparation in modified natural cycle (mNC) frozen embryo transfer (FET) cycles: the FET OPTIMIZING randomised controlled trial. Hum Reprod 2024a;39:deae108.237.

[hoaf058-B57] Saupstad M , Bergenheim SJ, Bogstad JW, Petersen MR, Klajnbard A, Prætorius L, Freiesleben NLC, Englund AL, Løkkegaard ECL, Knudsen UB et al Progesterone concentrations on blastocyst transfer day in modified natural cycle frozen embryo transfer cycles. Reprod Biomed Online 2024b;49:103862. 38735231 10.1016/j.rbmo.2024.103862

[hoaf058-B58] Schiffer L , Adaway JE, Baranowski ES, Arlt W, Keevil BG. A novel high-throughput assay for the measurement of salivary progesterone by liquid chromatography tandem mass spectrometry. Ann Clin Biochem 2019;56:64–71. 29792048 10.1177/0004563218780904

[hoaf058-B59] Tavaniotou A , Devroey P. Effect of human chorionic gonadotropin on luteal luteinizing hormone concentrations in natural cycles. Fertil Steril 2003;80:654–655. 12969719 10.1016/s0015-0282(03)00789-1

[hoaf058-B60] Tournaye H , Sukhikh GT, Kahler E, Griesinger G. A Phase III randomized controlled trial comparing the efficacy, safety and tolerability of oral dydrogesterone versus micronized vaginal progesterone for luteal support in in vitro fertilization. Hum Reprod 2017;32:1019–1027. 28333318 10.1093/humrep/dex023 PMC5400051

[hoaf058-B61] von Versen-Höynck F , Griesinger G. Should any use of artificial cycle regimen for frozen-thawed embryo transfer in women capable of ovulation be abandoned: yes, but what’s next for FET cycle practice and research? Hum Reprod 2022;37:1697–1703. 35640158 10.1093/humrep/deac125

[hoaf058-B62] von Versen-Höynck F , Narasimhan P, Selamet Tierney ES, Martinez N, Conrad KP, Baker VL, Winn VD. Absent or excessive corpus luteum number is associated with altered maternal vascular health in early pregnancy. Hypertension 2019;73:680–690. 30636549 10.1161/HYPERTENSIONAHA.118.12046 PMC6378337

[hoaf058-B63] Zaat T , de Bruin JP, Goddijn M, van Baal M, Benneheij S, Brandes M, Broekmans F, Cantineau A, Cohlen B, van Disseldorp J et al Home-based monitoring of ovulation to time frozen embryo transfers in the Netherlands (Antarctica-2): an open-label, nationwide, randomised, non-inferiority trial. Lancet 2023;402:1347–1355. 37678290 10.1016/S0140-6736(23)01312-0

[hoaf058-B64] Zhang Y , Fu X, Gao S, Gao S, Gao S, Ma J, Chen ZJ. Preparation of the endometrium for frozen embryo transfer: an update on clinical practices. Reprod Biol Endocrinol 2023;21:52. 37291605 10.1186/s12958-023-01106-5 PMC10249325

