# Supplementary material for: Live birth rates are unrelated to sex-steroid levels on ET day in a dydrogesterone-based ‘programmed-ovulatory FET’ protocol: a multi-centric prospective cohort study
Source: Hum Reprod Open. 2025 Sep 15;2025(4):hoaf058. doi: 10.1093/hropen/hoaf058 (PMC12598663; doi:10.1093/hropen/hoaf058)
Supplement: hoaf058_Supplementary_Data [file hoaf058_supplementary_data.docx]

**Supplementary Table S1 Patient demographics by clinical pregnancy status**

|  | Overall (n=559) | No Pregnancy (n=377) | Pregnancy (n=182) |
| --- | --- | --- | --- |
| Age at follicular puncture (years) | 34.2 (4.3) | 34.6 (4.3) | 33.5 (4.2) |
| Patients’ height (cm) | 168 (8.1) | 168 (8.8) | 168 (6.5) |
| Patients’ weight (kg) | 69.7 (14.6) | 68.3 (14.0) | 72.7 (15.4) |
| Body mass index (kg/m²) | 24.7 (4.9) | 24.1 (4.5) | 25.8 (5.4) |
| Duration of infertility (months) | 38.2 (24.6) | 39.1 (24.5) | 36.4 (24.7) |
| Caucasian, n (%) | 516 (92.3%) | 346 (92.0%) | 170 (93.4%) |
| Eumenorrheic, n (%) | 406 (72.6%) | 273 (73.0%) | 133 (73.1%) |
| AMH (ng/ml) | 3.0 (2.4) | 2.9 (2.3) | 3.2 (2.6) |
| Smoking, n (%) | 54 (9.7%) | 34 (9.0%) | 20 (11.0%) |
| Polycystic ovarian syndrome, n (%) | 31 (5.5%) | 19 (5.0%) | 12 (6.6%) |
| Endometriosis, n (%) | 78 (14.0%) | 56 (14.9%) | 22 (12.1%) |
| Lead follicle size at last monitoring (mm) | 17.2 (2.2) | 17.2 (2.1) | 17.1 (2.4) |
| EMT at last monitoring (mm) | 9.2 (2.1) | 9.2 (2.1) | 9.4 (2.1) |
| LH at last monitoring (IU/l) | 19.2 (20.2) | 18.7 (18.3) | 20.1 (23.8) |
| P at last monitoring (pg/ml) | 0.37 (0.29) | 0.38 (0.30) | 0.36 (0.26) |
| Duration until DYD initation after last monitoring (days) | 0.52 (0.78) | 0.52 (0.77) | 0.52 (0.81) |
| SET, n (%) | 485 (86.8) | 324 (85.9) | 161 (88.5) |
| DET or higher, n (%) | 74 (13.2) | 53 (14.1) | 21 (11.5) |

Demographics of the analyzed patient population with stratification for clinical pregnancy achievement; shown are mean and standard deviations or number and proportions. AMH, anti-Müllerian hormone; SET, single embryo transfer; DET, double embryo transfer; *missing values n=3.

**Supplementary Table S2 Ratios of geometric means of hormone levels at embryo transfer**

|  | **ratio** | **95%**  **confidence**  **interval** | |
| --- | --- | --- | --- |
| **Progesterone between FET days 4/5 and days 2/3** | 2.88 | 2.48 | 3.35 |
| **Estradiol between FET days 4/5 and days 2/3** | 1.39 | 1.27 | 1.53 |
| **DYD between FET days 4/5 and days 2/32/3** | 1.16 | 1 | 1.34 |
| **DHD between FET days 4/5 and days 2/3** | 1.14 | 1 | 1.3 |
| **Progesterone (ng/ml) by LH threshold (12.6 IU/L) at last follicular phase monitoring** | 1.36 | 1.19 | 1.56 |
| **Progesterone (ng/ml) by Follicular size threshold (16mm)** | 1.13 | 0.95 | 1.34 |
| **Estradiol (pg/ml) by LH threshold (12.6 IU/L) at last follicular phase monitoring** | 0.91 | 0.85 | 0.99 |
| **Estradiol (pg/ml) Follicular size threshold (16mm)** | 0.96 | 0.87 | 1.06 |

FET, frozen-thawed embryo transfer; DYD, dydrogesterone; DHD, 20α-dihydrodydrogesterone.

**Supplementary Table S3 Observed and inferred hormone value distributions on different days of FET.**

| **Observed quantiles of hormone concentrations on different days of FET** | | | | | | | | | | | | | | | | |
| --- | --- | --- | --- | --- | --- | --- | --- | --- | --- | --- | --- | --- | --- | --- | --- | --- |
| **Day of FET** | **DYD (ng/ml)** | | | | | **DHD (ng/ml)** | | | | | | **Progesterone (ng/ml)** | | | | |
|  | **5%** | **25%** | **50%** | **75%** | **95%** | **5%** | **25%** | **50%** | **75%** | **95%** | **5%** | | **25%** | **50%** | **75%** | **95** |
| **2** | 0.29 | 0.61 | 0.96 | 1.39 | 2.60 | 10.79 | 19.14 | 30.94 | 45.06 | 64.39 | 0.50 | | 1.24 | 2.96 | 5.73 | 14.01 |
| **3** | 0.40 | 0.64 | 0.91 | 1.56 | 3.35 | 14.97 | 23.24 | 30.69 | 45.12 | 82.57 | 0.64 | | 1.85 | 3.62 | 7.38 | 14.64 |
| **4** | 0.42 | 0.84 | 1.06 | 1.45 | 3.12 | 15.09 | 25.87 | 34.41 | 50.12 | 84.34 | 3.69 | | 4.39 | 6.63 | 8.66 | 15.03 |
| **5** | 0.43 | 0.71 | 1.11 | 1.76 | 3.53 | 15.06 | 25.03 | 36.46 | 57.22 | 93.33 | 2.43 | | 6.67 | 8.9 | 11.92 | 18.03 |

| **Inferred quantiles of hormone concentrations on different days of FET assuming lognormal distributions.** | | | | | | | | | | | | | | | |
| --- | --- | --- | --- | --- | --- | --- | --- | --- | --- | --- | --- | --- | --- | --- | --- |
| **Day of FET** | **DYD (ng/ml)** | | | | | **DHD (ng/ml)** | | | | | **Progesterone (ng/ml)** | | | | |
|  | **5%** | **25%** | **50%** | **75%** | **95%** | **5%** | **25%** | **50%** | **75%** | **95%** | **5%** | **25%** | **50%** | **75%** | **95** |
| **2** | 0.33 | 0.62 | 0.97 | 1.51 | 2.86 | 11.84 | 20.53 | 30.09 | 44.12 | 76.49 | 0.36 | 1.15 | 2.57 | 5.72 | 18.10 |
| **3** | 0.35 | 0.66 | 1.02 | 1.59 | 3.01 | 13.22 | 22.7 | 33.06 | 48.13 | 82.64 | 0.65 | 1.75 | 3.49 | 6.97 | 18.84 |
| **4** | 0.41 | 0.74 | 1.12 | 1.69 | 3.05 | 14.42 | 25.4 | 37.64 | 55.77 | 98.21 | 3.18 | 4.92 | 6.67 | 9.03 | 13.98 |
| **5** | 0.37 | 0.72 | 1.15 | 1.81 | 3.50 | 12.29 | 23.42 | 36.66 | 57.39 | 109.4 | 2.57 | 5.05 | 8.08 | 12.94 | 25.45 |

FET, frozen-thawed embryo transfer; DYD, dydrogesterone; DHD, 20α-dihydrodydrogesterone.

**Supplementary Table S4 Hormone levels at embryo transfer as predictors of live birth: logistic regression analysis**

|  | **Odds ratio** | **95%-confidence** | **interval** |
| --- | --- | --- | --- |
| **P** | 1.023 | 0.979 | 1.068 |
| **E2** | 1 | 0.9989 | 1.001 |
| **DYD** | 0.8823 | 0.7182 | 1.068 |
| **DHD** | 0.9929 | 0.9846 | 1.001 |
| **P adj. for E2** | 1.052 | 0.9562 | 1.16 |
| **E2 adj. for P** | 1 | 0.9988 | 1.002 |
| **P-E2 interaction** | 0.9999 | 0.9997 | 1 |
| **P adj. for DYD** | 1.037 | 0.9586 | 1.121 |
| **DYD adj. for P** | 0.9697 | 0.6103 | 1.479 |
| **P-DYD interaction** | 0.9876 | 0.9365 | 1.043 |
| **P adj. for DHD** | 1.024 | 0.9357 | 1.119 |
| **DHD adj. for P** | 0.9932 | 0.9746 | 1.011 |
| **P-DHD interaction** | 1 | 0.9979 | 1.002 |
| **DYD adj. for E2** | 0.9925 | 0.6376 | 1.523 |
| **E2 adj. for DYD** | 1 | 0.9986 | 1.002 |
| **DYD-E2 interaction** | 0.9996 | 0.9984 | 1.001 |

E2, estradiol; P, progesterone; DYD, dydrogesterone; DHD, 20α-dihydrodydrogesterone.

**Supplementary Figure S1 Overview flowchart of all patients enrolled in the study.** Flowchart depicting patients undergoing FET during the study period. The denominator for the current analysis is 559 patients on PO-FET protocol and hormone level assessments on day of FET. FET, frozen-thawed embryo transfer; PO-FET, progesterone-optimized frozen embryo transfer


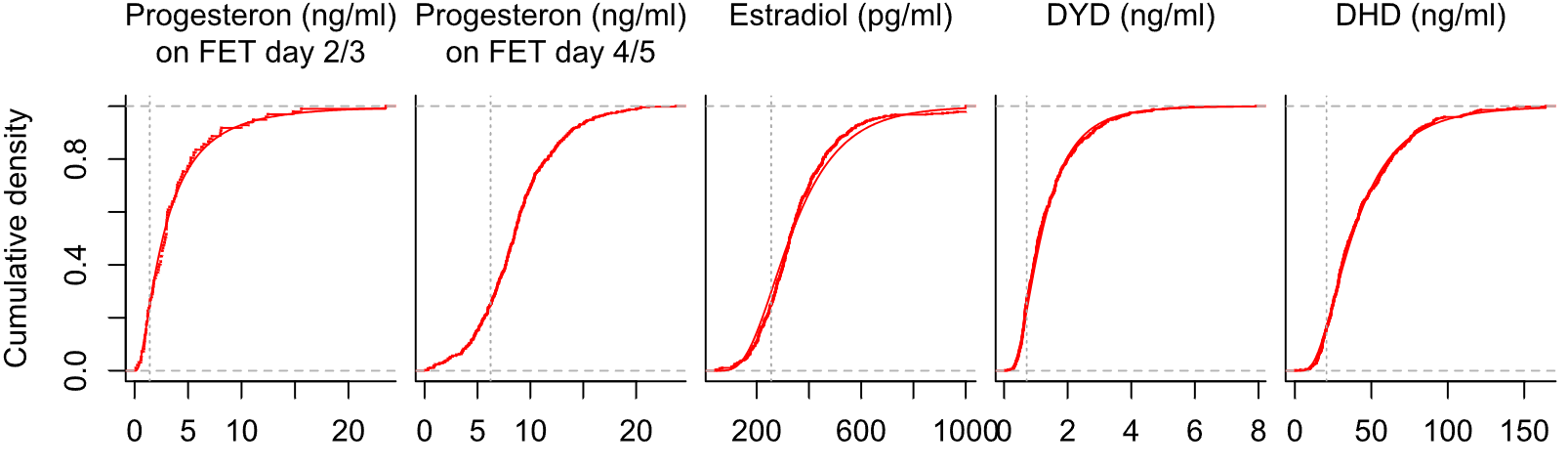


**Supplementary Figure S2 Distribution of hormone levels on day of FET with lower quartile references** Empirical and lognormal hormone value distributions on day of FET. Vertical reference lines are lower quartiles at embryo transfer previously described, or, for estradiol and progesterone, in the present data; for progesterone, separately by day of FET. FET, frozen-thawed embryo transfer; DYD, dydrogesterone; DHD, 20α-dihydrodydrogesterone.

**Supplementary Figure S3 Interplay between progesterone, follicular size, LH, and E2 by FET day with threshold stratification**


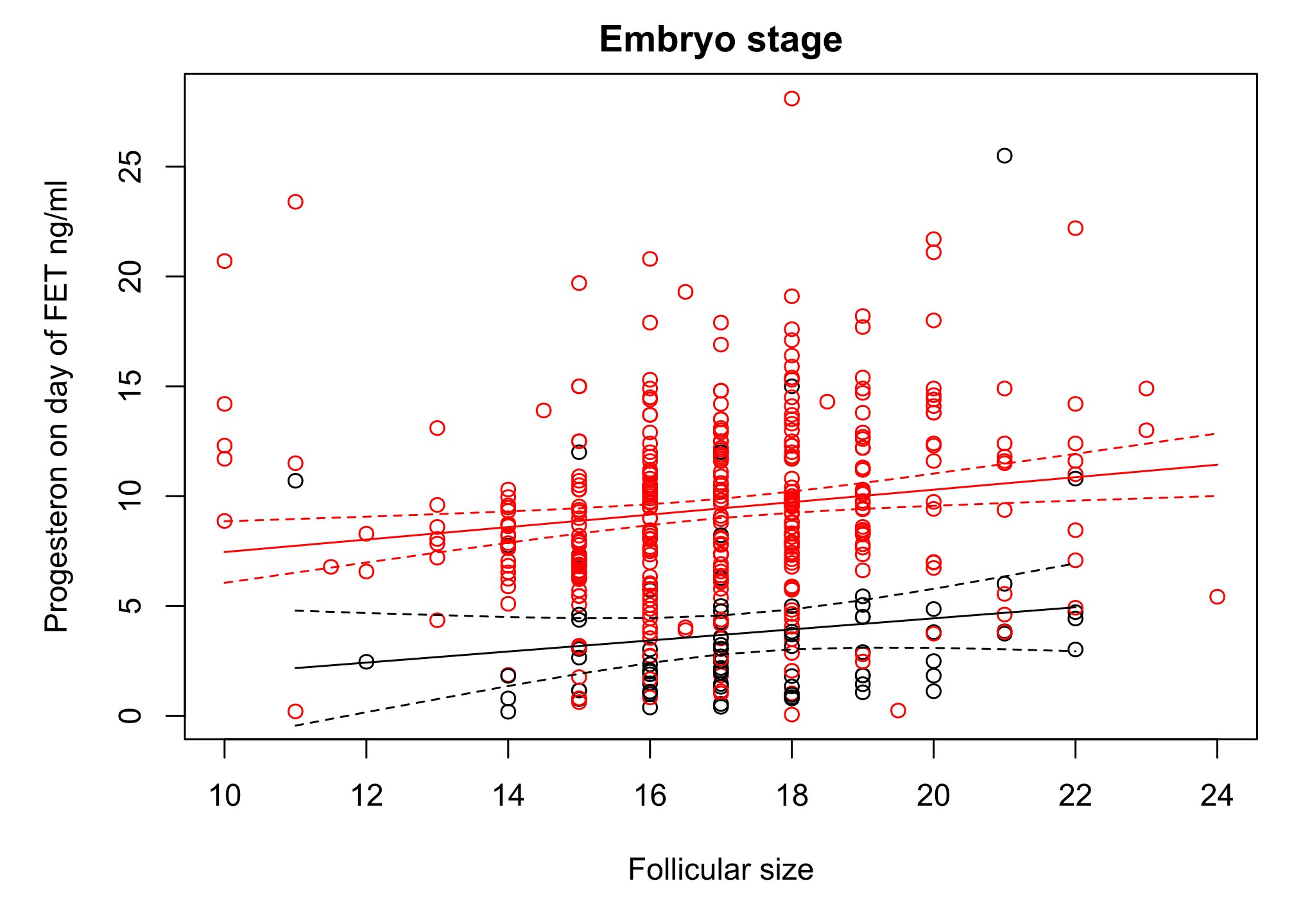


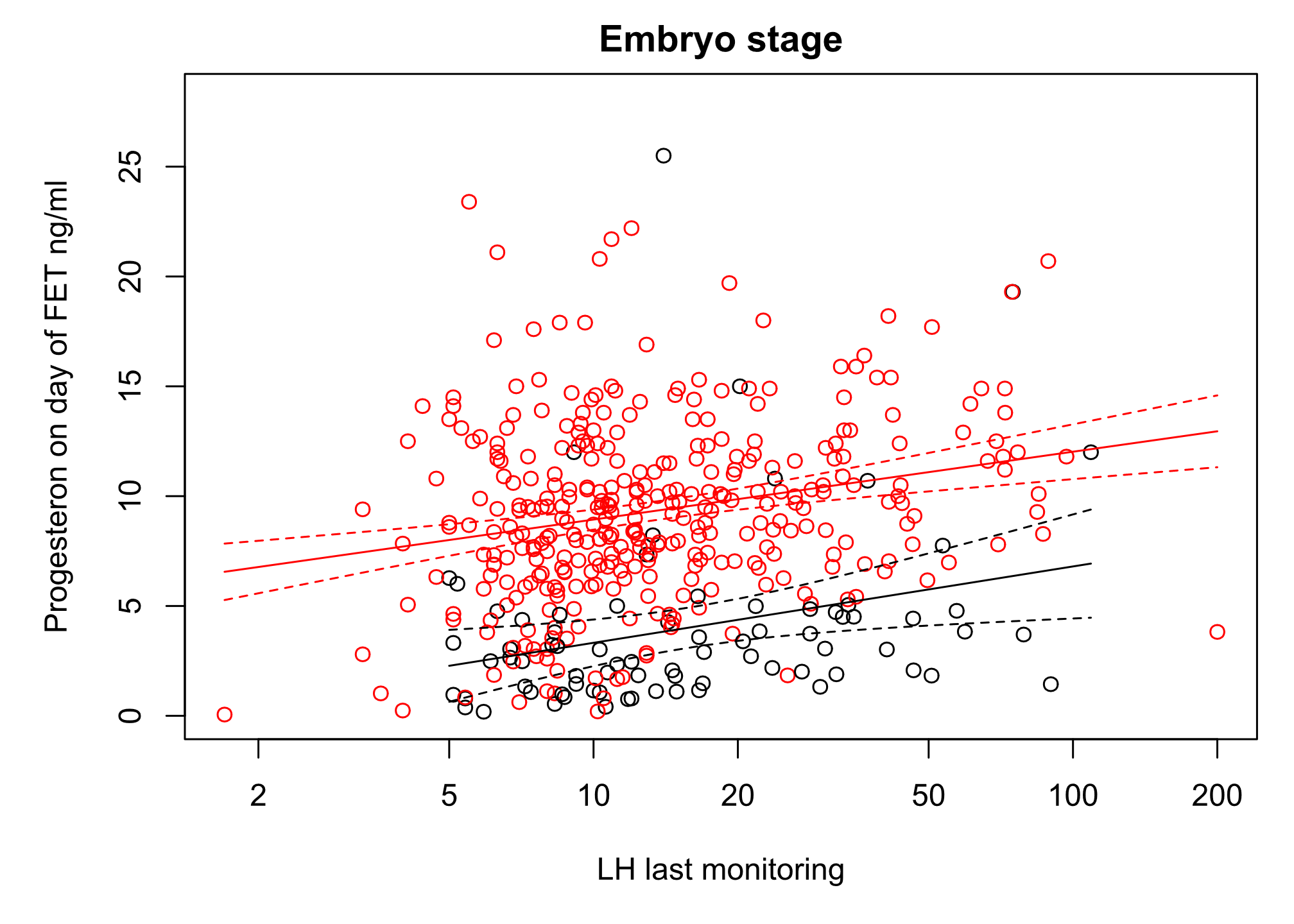


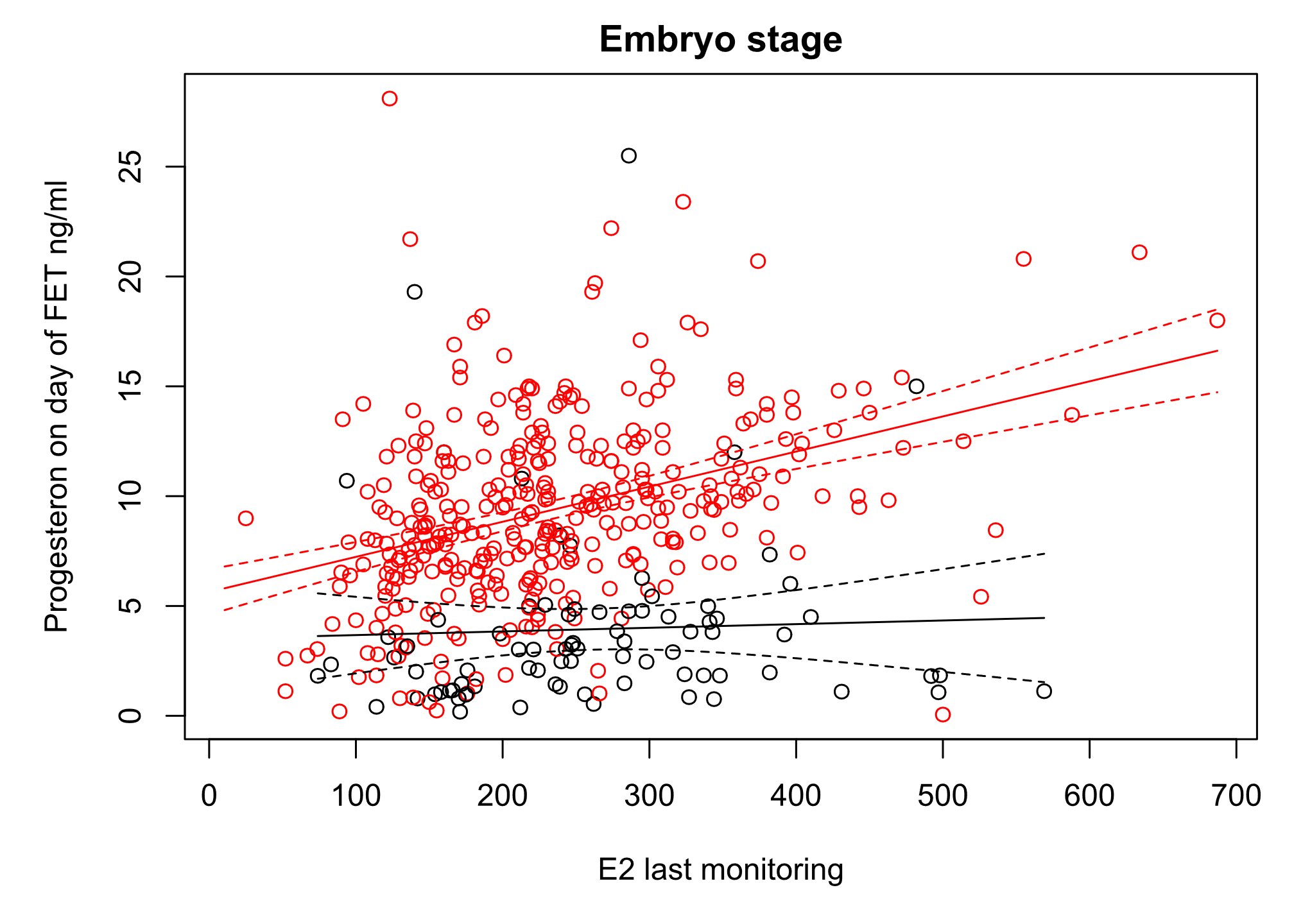


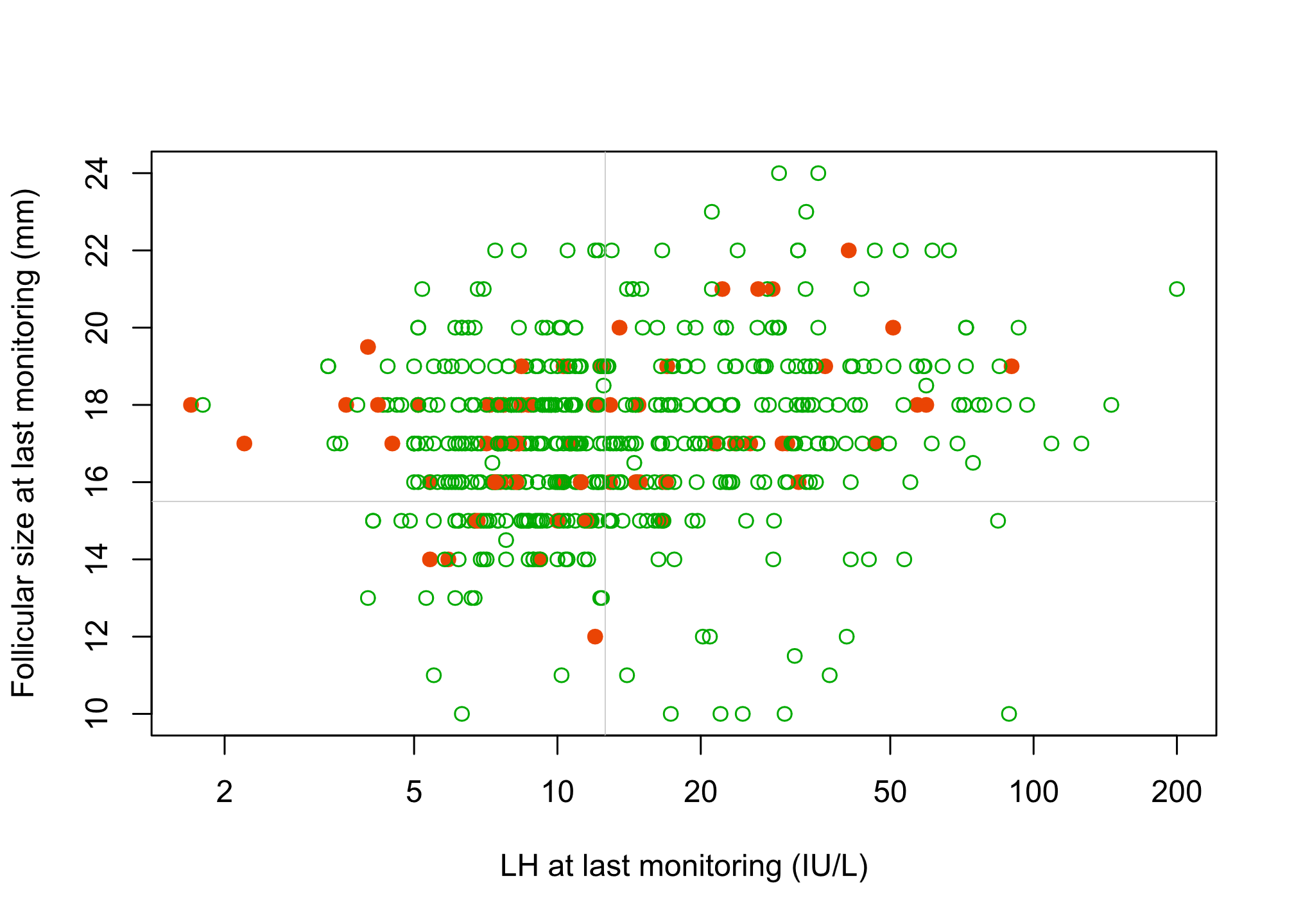


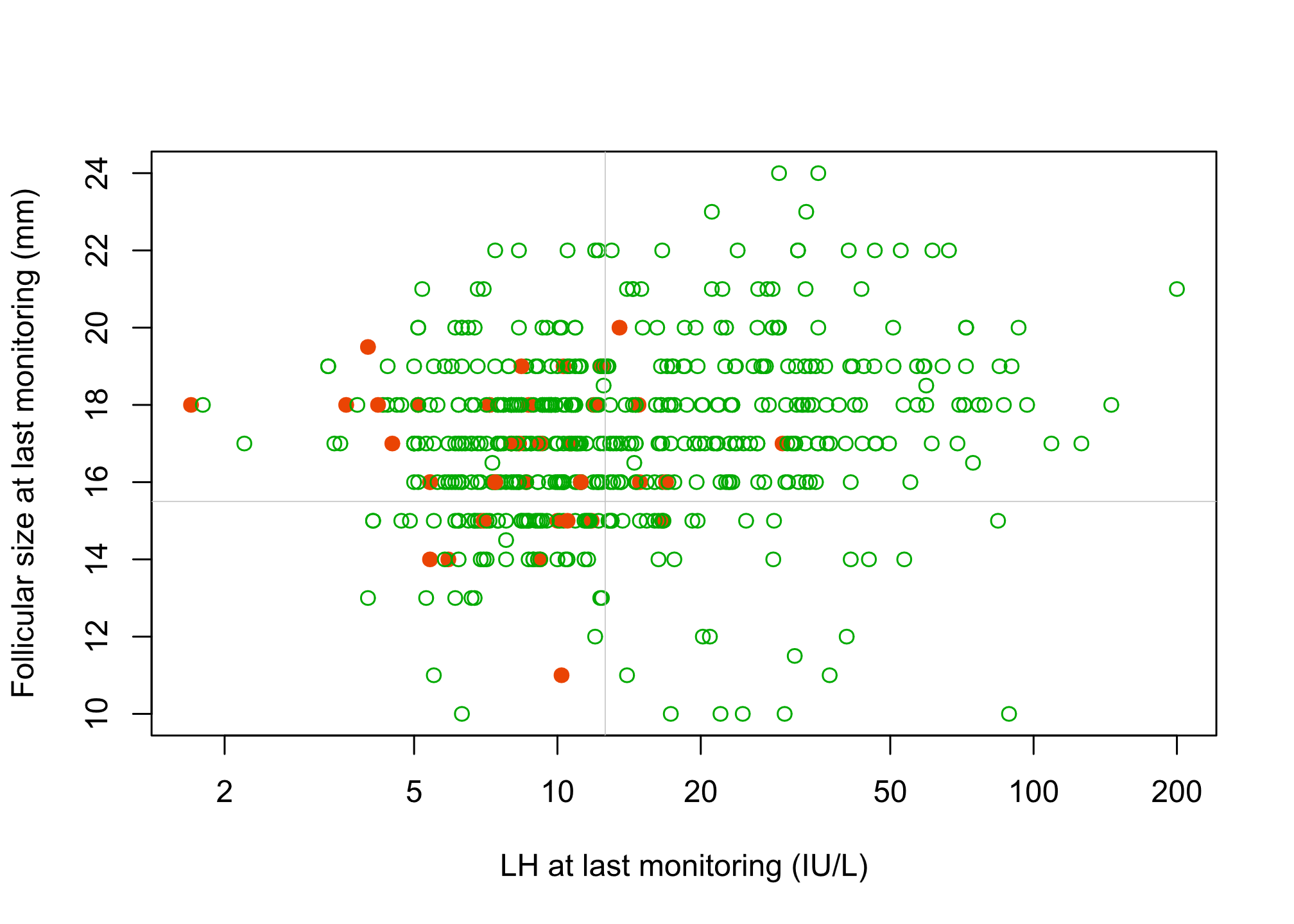


Scatter plots illustrating the relationship between serum progesterone levels at the time of frozen embryo transfer (FET) and follicular size, serum LH, and serum E2 levels at the last monitoring, with trend lines, 95% confidence intervals, for FET at day 2/3 (black) and at day 4/5 (red). The last scatterplots show progesterone at day of FET ≥ 1.5 ng/ml (green circles) and < 1.5 ng/ml (orange dots) by follicular size and LH levels at last monitoring with thresholds 16 mm and 12.6 IU/L indicated. While the last-but-one plot shows all observations, the last shows just cases with FET day 4/5. E2, estradiol.


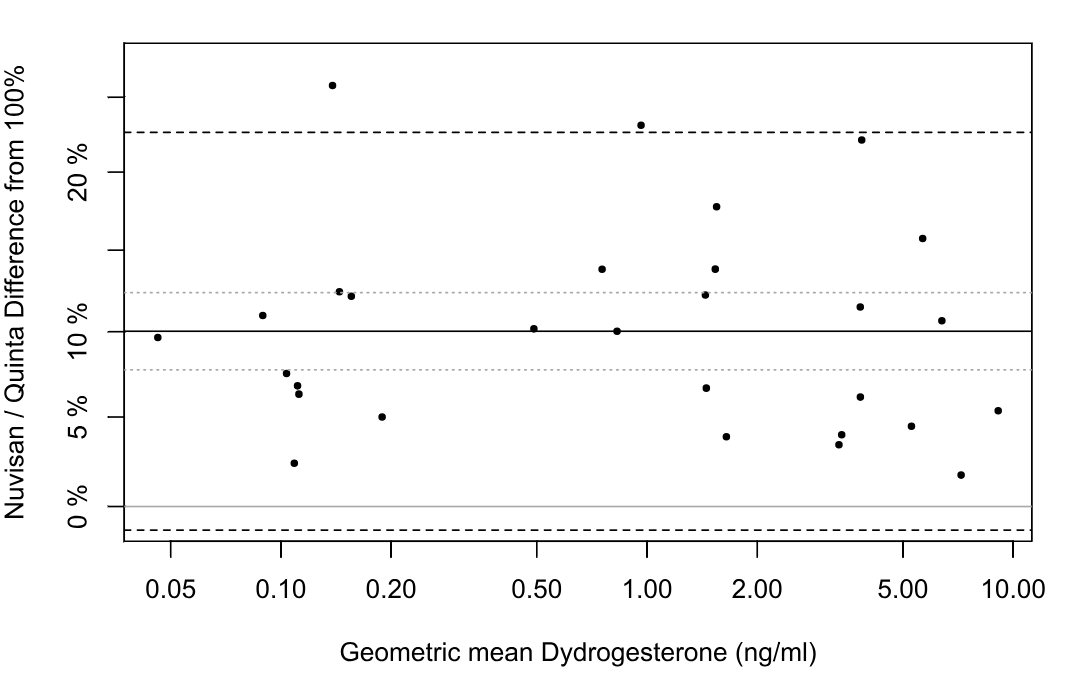

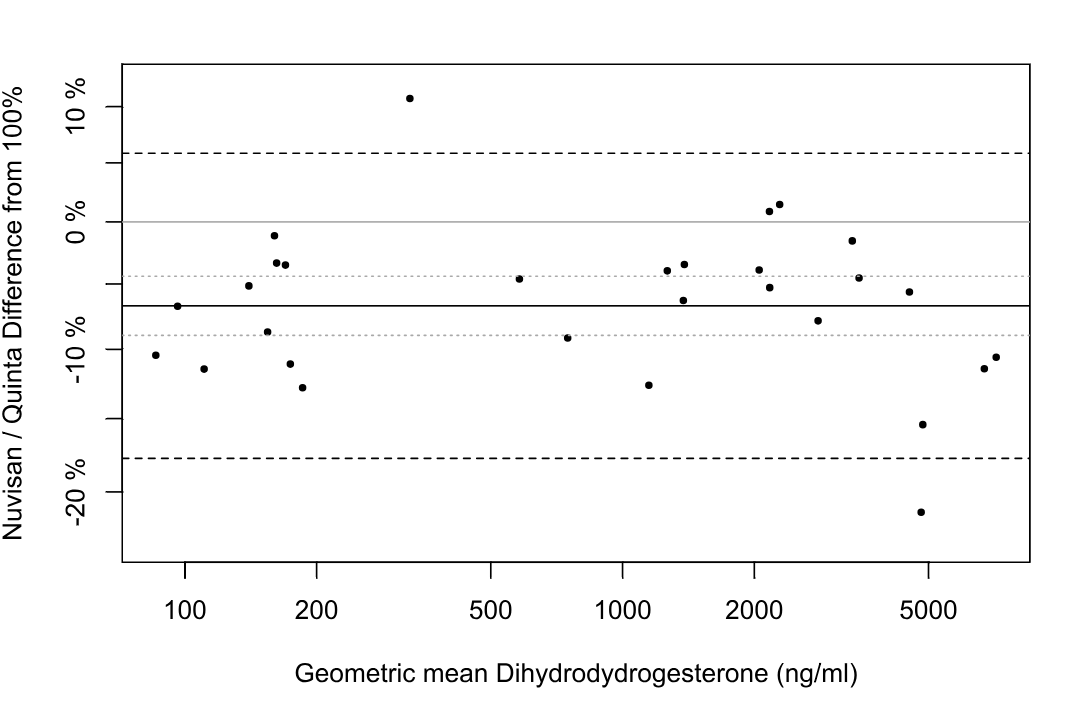


b)b)

a)

**Supplementary Figure S4 Bland-Altmann analysis and hormone threshold classification for DYD and DHD.** Bland-Altman analysis comparing measurement methods for DYD (a) and DHD (b) using two different HPLC/MS/MS platforms. DYD showed a positive bias of 10.03% (95% ci: 7.74 to 12.37) with limits of agreement from -1.28% to 22.62%. DHD showed a negative bias of -6.7% (95% ci: -8.96 to -4.39) with limits of agreement from -17.75% to 5.84%. Visual inspection of Bland-Altmann plots indicates good agreement across hormone concentration ranges. Previously established thresholds for DYD (0.71 ng/ml) and DHD (20.67 ng/ml) were used to classify patients into low vs. normal-high groups (Neumann et al., 2022). DYD, dydrogesterone; DHD, 20α-dihydrodydrogesterone.
